# Mapping Microplastics in Humans: Analysis of Polymer Types, and Shapes in Food and Drinking Water—A Systematic Review

**DOI:** 10.3390/ijms25137074

**Published:** 2024-06-27

**Authors:** Alena Vdovchenko, Marina Resmini

**Affiliations:** Department of Chemistry, School of Physical and Chemical Sciences, Queen Mary University of London, London E1 4NS, UK; a.vdovchenko@qmul.ac.uk

**Keywords:** microplastic, nanoplastic, food contamination, environmental health, plastic pollution

## Abstract

Microplastics (MPs) pervade the environment, infiltrating food sources and human bodies, raising concerns about their impact on human health. This review is focused on three key questions: (i) What type of polymers are humans most exposed to? (ii) What are the prevalent shapes of MPs found in food and human samples? (iii) Are the data influenced by the detection limit on the size of particles? Through a systematic literature analysis, we have explored data on polymer types and shapes found in food and human samples. The data provide evidence that polyester is the most commonly detected polymer in humans, followed by polyamide, polyurethane, polypropylene, and polyacrylate. Fibres emerge as the predominant shape across all categories, suggesting potential environmental contamination from the textile industry. Studies in humans and drinking water reported data on small particles, in contrast to larger size MPs detected in environmental research, in particular seafood. Discrepancies in size detection methodologies across different reports were identified, which could impact some of the discussed trends. This study highlights the need for more comprehensive research on the interactions between MPs and biological systems and the effects of MPs on toxicity, together with standardised analytical methodologies to accurately assess contamination levels and human exposure. Understanding these dynamics is essential for formulating effective strategies to mitigate the environmental and health implications of MP pollution.

## 1. Introduction

Plastic pollution is a global challenge, with numbers providing evidence of its magnitude. The Birmingham Plastics Network recently reported that humanity has produced over 10 billion tonnes of plastic, 80% of which has been discarded [1]. When plastic waste is not disposed of correctly, or recycled, it escapes into the environment, where it is exposed to sunlight, heat, and humidity, often resulting in fragmentation and leading to the formation of microplastics (MPs)—generally defined as small plastic fragments measuring below 5 mm in size [2]. As these fragments start to contaminate the environment, they predictably infiltrate water sources and the food chain.

The pathways through which MPs infiltrate the food chain have been extensively explored and reported in the literature. Firstly, MPs enter the environment through direct consumption by aquatic and terrestrial organisms, such as fish [3], seafood [4], livestock, and poultry [5,6]. Secondly, during food processing, contamination can occur via conveyor belts or clothing of food industry workers [7], or through water contamination facilitated by plastic pipes [8], or storage containers at drinking water treatment plants [9]. Moreover, studies have highlighted potential contamination routes through the use of plastic cutting boards [10,11]. Additionally, airborne MPs can be deposited on food sources during various stages of production and handling [12]. Finally, packaging plays a significant role, with MPs entering food products through various vessels like teabags [13], plastic water bottles [14], cups for hot beverages [15], or through microwaving food containers [16]. Collectively, the literature data underscore the multifaceted ways in which plastic pollution infiltrates our environment and ultimately, our food supply.

The presence of MPs in the food chain has raised significant concerns regarding their potential impact on human health, leading to a multitude of studies focusing on toxicological assessment. These studies encompass various approaches, including analyses of isolated biomolecules to evaluate MP interactions with enzymes [17,18], investigations using cell lines [19], research involving animals [20], and the identification of MPs in samples from humans—including both healthy individuals and patients with various diseases [21]. Several studies have reported concerning findings associated with MP exposure, such as inflammation [22], changes in the gut microbiome composition [23], effects on reproductive health [24], and potential links with vascular pathologies [25,26].

While some researchers have investigated human exposure to MPs, their work has primarily focused on MPs in general, without differentiating the materials based on their physico-chemical characteristics. For instance, the study conducted by Cox et al. estimated that the annual consumption of MPs through food and drinking water falls within the range of 39,000 to 52,000 particles, with some variations depending on age and sex [27]. However, this study did not include any discussion on the chemical composition, shape, or size of MPs. While quantifying the annual MP exposure through food is crucial, the qualitative characterisation of nanoparticles is equally important. There is still a significant knowledge gap, as the properties of MPs to which humans are exposed have not yet been systematically evaluated.

**Figure 1:**
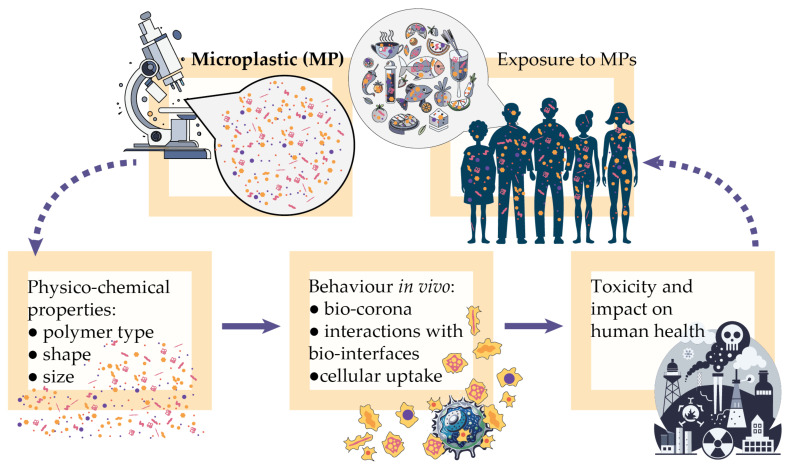
Schematic representation of the correlation between physico-chemical properties of MPs, their behaviour in biological systems, and impact on human health.

The physico-chemical properties of MPs play a crucial role in determining their behaviour in biological systems (Figure 1). Upon entering the biological environment, micro- and nanoparticles can interact with a variety of molecules, resulting in the formation of a bio-corona composed of proteins, lipids, or other organic matter adsorbed at the particle interface [28]. Bio-corona formation is influenced by factors such as particle charge, surface roughness, and surface hydrophobicity [29]. In turn, the bio-corona will alter the MPs’ morphology and properties, significantly affecting their cellular uptake, interactions with different types of membranes, and overall behaviour at the bio-interface [17,30,31]. The shape of polymeric micro- and nanoparticles is particularly important in the context of behaviour in the biological systems, as it can influence tissue penetration and interactions with bio-interfaces [32,33]. Therefore, the evaluation of both the morphology and physico-chemical properties of MPs to which humans are exposed is of paramount importance, as it can significantly impact the toxicity of materials and, consequently, human health. 

In this study, by utilising a systematic analysis of the literature published up to April 2024, we aimed to evaluate data on the polymer type, shape, and size of MPs that have been found in food sources and human samples. The work centred around answering three important questions: (i) What type of polymers are humans most exposed to? (ii) What are the prevalent shapes of MPs found in food and human samples? (iii) Are the data influenced by the detection limit on the size of particles? The focus of the analysis was on the correlation between MPs found in humans and those found in food and drinking water sources. To effectively analyse this correlation, we excluded inhalation and MPs found in lung tissues. While MPs are hypothesised to potentially enter in systemic circulation through the lungs, it is still challenging to differentiate the proportion of MPs that are airborne from those that enter the human body through food. The results are discussed within the context of their toxicological effects and implications for human health, focusing particularly on interactions between MPs and biosystems at the molecular level. This work aimed to fill the knowledge gap regarding the most common polymer types and shapes occurring in food sources and humans and is expected to impact future studies assessing the toxicological effects of MP pollution on human health. Furthermore, it can inform policy-makers, guide public health strategies, and influence environmental management practices.

## 2. Methods

The data collection process involved a systematic literature analysis utilising three comprehensive databases: PubMed (accessed on 20 March 2024), Web of Science (accessed 27 March 2024), and Scopus (accessed on 31 March 2024). A thorough search strategy was implemented, encompassing six distinct categories, namely seafood invertebrates, fish, drinking water, meat, plant-based food, and humans. Each category was searched independently within the databases. The search focused exclusively on original articles published in English with full-text accessibility, meaning the complete text was available either through open access or via institutional subscriptions, while excluding literature reviews, conference abstracts, and book chapters. To optimise the search outcomes, a wildcard (*) was incorporated with the terms “microplastic” and “human.” Table 1 provides a summary of the search terms employed for each category.

**Figure 2:**
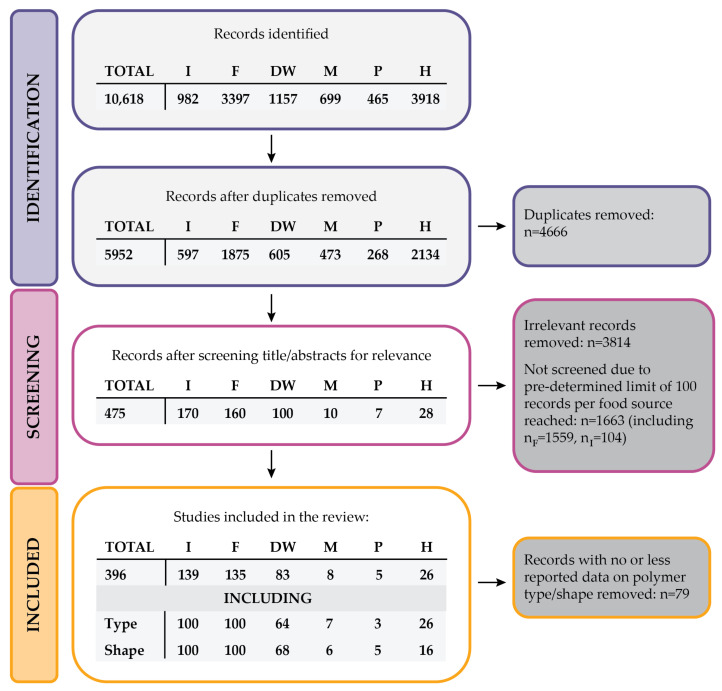
Flow diagram describing the process of evidence collection for the literature analysis; numbers represent the total number of records (TOTAL) and the number of publications in each food group identified at different stages of evidence collection. Abbreviations used for food groups: I—seafood: invertebrates, F—fish, DW—drinking water, M—meat, P—plant-based food, H—humans.

The flow diagram illustrating the process of identifying evidence used for data analysis is presented in Figure 2. Initially, the search yielded 10,618 pieces of evidence across all analysed groups, which were subsequently refined to 5952 unique items after eliminating duplicates. The screening process commenced with a manual review of titles and abstracts to exclude irrelevant publications. Irrelevant publications predominantly fell into one of the following categories: (1) model studies (in vitro, in situ, or in silico), (2) analyses of MPs in non-edible species, and (3) studies examining MPs in the environment (sea, freshwater, air, soil) without a focus on food sources or human samples. The full list of studies used for the analysis of MPs in food sources is provided in the Supplementary Materials.

For two groups—I and F—a limit of 100 publications with relevant data on polymer type and MP shape was determined. The selection of publications was systematic, based on the order of appearance in the search results from the three databases used. This limit ensured a manageable dataset for evaluating the ratio of common polymer types and MP shapes found in these food sources. This approach strikes a balance between inclusivity and feasibility, allowing for a thorough examination of trends while maintaining the depth of analysis necessary for meaningful insights.

Therefore, upon identifying 100 publications with adequate data quality for both polymer type and MP shape analysis in the I and F groups, further manual screening of remaining publications within those groups was deemed unnecessary. However, for the remaining four groups (DW, M, P, and H), the 100-publication limit was not reached even after analysing all the available publications.

After the initial screening process, the full texts of the selected publications were analysed to extract the following data: the occurrence of MPs based on their chemical structure, MPs’ shapes, and the minimum size of MPs analysed. During the extraction of data, it was assumed that the data presented in the published literature are valid and accurate and the publications were not assessed for data quality or whether control measures were used. Since a variety of terms are used in the literature to describe MP shapes, it was decided to group them into five categories, summarised in Table 2.

When data on MP type/shape were presented as percentages alongside the total number of analysed particles, the data were standardised by converting percentages to the number of occurrences. However, publications were excluded from the dataset if they did not provide information on the total number of analysed MPs or if details regarding MP polymer type/shape were absent. As a result, a total of 79 publications with no or less data were removed from the analysis.

It is important to note that while some publications provided information on both polymer type and MP shape, others reported only one characteristic. Consequently, the total number of publications used per group may not align with the number used, specifically for MP type/shape analysis.

## 3. Results and Discussion

### 3.1. Critical Analysis of Human Exposure to MPs through Food and Drinking Water

#### 3.1.1. Description of Data Sources

Before discussing the specifics of the data, it is essential to provide some details regarding the breadth and diversity of studies included in the analysis. The following food groups were selected—seafood invertebrates, fish, drinking water, meat, and plant-based food, each contributing to the evaluation of MP contamination within various dietary sources. The diversity of food sources considered in the analysis is summarised in Table 3. Studies examining MP contamination in species intended for human consumption formed the basis of our database. Notably, the study did not prioritise any specific dietary pattern, encompassing a wide range of food sources reflective of various regions and culinary preferences. For instance, species such as snails or sea cucumbers, typical to certain geographical areas, were also included in the analysis. In addition to the primary food groups mentioned, MPs were also detected in other food items such as tea bags [34], baby food [35], salt [36], sugar [37], honey, beer, milk, etc. [38]; however, these studies were not included in our analysis. The exclusion is justified by the limited number of studies available on these food sources and their comparatively lower consumption rates compared to the highlighted groups.

The disparity in publication numbers across different food groups is evident from the data summary, with aqueous environments such as seafood and freshwater species, as well as drinking water being clearly prevalent. Conversely, research on MPs in meat (*N* = 8) and plant-based food (*N* = 5) is relatively recent and emerging. Consequently, while these studies have been integrated into analysis, their limited representation suggests a need for cautious interpretation and discussion.

The data analysed as part of this work focused on the occurrence of MPs in humans, without differentiating between studies on healthy individuals and those with a health condition. Table 4 provides a summary of the publications that found MPs in humans, detailing the number of individuals, their health status, and the quantity and minimal size of MPs identified. 

The data clearly indicate that research on MP exposure in humans predominantly focuses on the gastrointestinal tract, the vascular system, and reproductive health, including the exposure of new-borns to MPs. Notably, selected publications exhibit considerable variation in the reported number of MPs per patient, organ, and tissue. Such disparities are likely due to differences in the methodologies employed for both sample preparation and MP analysis, as previously suggested [64,65]. This review used the data on occurrence of MP particles rather than concentration, to reduce the impact of methodological differences and provide a more robust analysis of the prevalence of MPs in human samples.

#### 3.1.2. Human Exposure to MPs: Evaluation of Polymer Chemistry

In this section, we present the available data to try to address a pivotal question: is there a correlation between the global production of specific polymers and their occurrence as MPs in food sources and humans? The analysis of the available data was expected to allow the formulation of important predictions, together with suggestions for suitable countermeasures. It was decided to evaluate eight of the most prevalent plastic types—generic polyester (PES), polypropylene (PP), polyamide (PA), polyvinyl chloride (PVC), polyethylene (PE), polyacrylate (PAC), polystyrene (PS), and polyurethane (PU). These plastic types were identified based on their frequent mention in the literature across the 396 sources analysed as common MP pollutants. By prioritising the evaluation of these dominant MP types, we aimed to understand which polymer chemistry of MPs should be explored in more detail when evaluating the impact of MPs on human health and the environment.

**Figure 3:**
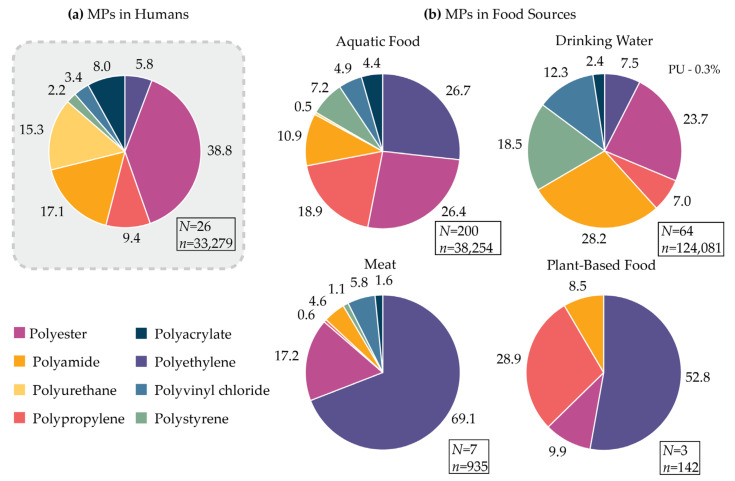
Ratio of the eight most common MP types found in human samples (**a**) and different food sources (**b**); number represents the percentage drawn from *n*; *N* = number of studies included in the analysis; *n*—total number of PES, PA, PU, PP, PAC, PE, PVC, and PS MPs identified.

The summary of our findings is presented in Figure 3. The results reveal that the five primary types of MPs detected in humans are PES (38.8%), PA (17.1%), PU (15.3%), PP (9.4%), and PAC (8.0%). The percentages reflect the prevalence among the eight most commonly found MP types; therefore, MPs with unconfirmed or uncommon chemical structures were excluded from the data analysis. Table 5 provides a concise overview of the estimated global production of the eight plastic types and offers examples of their common uses.

The data indicate that among the eight most prevalent plastic materials detected in humans, PES comprises 38.8%. PES stands out as one of the highest weights of global production, reaching 90.9 million tonnes, and finds extensive applications in everyday items such as food packaging, clothing, and home textiles (e.g., rugs, mattresses, and furniture materials). The presence of polyester in meat (17.2%) and plant-based foods (9.9%) may stem from contamination originating from packaging materials. Meanwhile, its presence in aquatic food (26.4%), and drinking water (23.7%) suggests the widespread contamination of water resources and high levels of environmental pollution with PES. Human exposure to polyester extends beyond food ingestion to include the inhalation of PES dust from textiles, as highlighted in a review by O’Brien et al. [68].

Among the eight analysed MP types, PA accounts for 17.1%, emerging as the second most prevalent MP found in human samples. Interestingly, the global production of PA (6.1 Mt) is one of the lowest among the studied materials, but data provide evidence of PA contamination in all analysed food sources. Notably, a high presence of PA (28.2%) is reported in drinking water, including bottled water [69], tap water [70], and purified water from drinking water treatment plants (DWTPs) [71]. The ubiquity of this contamination suggests that it may not solely originate from packaging but rather from environmental sources. Studies have documented the release of MPs from nylon (PA) fishing nets, contributing alongside other sources to the contamination of water resources [72,73]. Indeed, the ratio of PA in aquatic food is 10.9%, highlighting significant water resource pollution. In meat and plant-based foods, polyamide constitutes 4.6% and 8.5% of the analysed plastics, which are potentially contaminated during food processing through conveyor belts, the clothing of food industry workers, or packaging materials. Overall, PA emerges as a pervasive material; humans are exposed through the consumption of food and beverages, as well as through the inhalation of dust originating from textiles.

PU is identified as the third noted polymer type detected in human samples, comprising 15.3% of the total. Surprisingly, PU was found in relatively low quantities compared to other analysed polymers in aquatic food and drinking water (≤0.5%) and has not yet been recorded in meat and plant-based food. In human samples, PU was identified in vascular tissues [51], placenta [58], maternal fluid [60], and meconium of infants [58]. These findings are surprising and suggest that human exposure to PU may not primarily occur through food sources, but rather through alternative routes. Liu et al. suggested that the observed contamination of pregnant women and infants with PU primarily results from indoor dust, as PU is extensively used in textiles such as furniture, carpets, and clothing [58].

Among the eight plastic materials analysed, PP constitutes 9.4% of the MPs found in humans. With a global production of approximately 69.3 million tonnes, PP stands as the third most widely produced plastic material [67]. Notably, PP is prevalent in various sources, with concentrations ranging from 7% in drinking water, 18.9% in aquatic food, to a very high amount found in plant-based foods (28.9%), indicative of environmental contamination with this plastic type. In plant-based foods, a notable presence of PP was documented in vegetable oil in the study by Battaglini et al. [74]. However, pinpointing the exact source of this contamination poses challenges, as PP finds broad utility across all stages of vegetable oil production, including farming, transportation, vegetable processing, and packaging of the final product.

Among the analysed polymers, PAC emerges as the fifth most found type in humans, accounting for 8% of the identified MPs. Similar to PU, PAC does not dominate across various food sources, constituting 4.4% in aquatic food, 2.4% in drinking water, and 1.6% in meat, and it has not yet been reported in plant-based foods. In human samples, PAC has been detected in stool, vascular, and ocular tissues. Given its extensive application in cosmetics and coatings, the data can be used to hypothesise that human exposure to PAC may primarily originate from daily used products rather than food sources.

Other materials such as PE, PVC, and PS represent a smaller proportion of MPs found in human samples, accounting for 5.8%, 3.4%, and 2.2%, respectively. Among these, PE stands out due to its remarkably high global production value of 106.6 million tonnes, and it is frequently listed among the most commonly found polymers in the environment [67]. PE exhibits a widespread presence in food sources, representing 26.7% of MPs found in aquatic species, 7.5% in drinking water, 69.1% in meat, and 52.8% in plant-based foods. The contamination of food by PE can stem from various sources, including packaging [75], the use of cutting boards [10,11], and containers utilised for microwaving [16].

**Figure 4:**
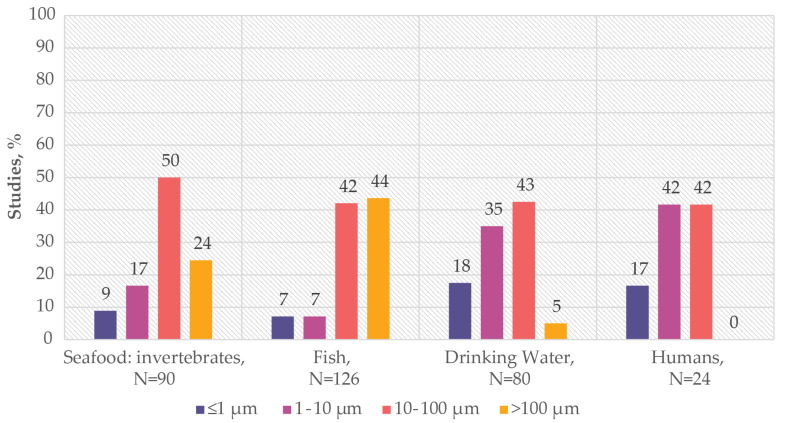
Minimum size of MPs analysed in different sample groups (seafood: invertebrates, fish, drinking water, humans); *N*—number of studies. Y axis represents the percentage of studies where the minimum size of analysed MPs belongs to the group ≤ 1 µm, 1–10 µm, 10–100 µm, >100 µm.

While data on the abundance of MP types in food sources and human samples give insight into human exposure to MPs, the values need to be analysed with caution due to the large variations in the methods used for the extraction and characterisation of MPs. Indeed, the lack of uniformity in protocols for MPs evaluation has been highlighted before [64,65,67]. One critical parameter that influences the reported data is the minimum size of MPs analysed. The smallest size identified in studies is often limited by the capabilities of the equipment and extraction techniques used. Depending on the size detection limit, researchers may initially look at different populations of MPs within a sample. To contextualise the results of MP occurrence in terms of analysed MP size, the publications used for this study were categorised into four groups based on the lowest size of MPs characterised: ≤1 µm, 1–10 µm, 10–100 µm, and >100 µm. This approach allowed us to consider the size range initially evaluated and to highlight discrepancies in size detection methodologies across different reports. The percentage of studies in each food category belonging to these size groups is presented in Figure 4. 

As the data show, in seafood invertebrates and fish samples, 74% and 86% of studies, respectively, analyse MPs with sizes above 10 µm. Specifically, in fish samples, the majority (44%) of studies focus solely on MPs above 100 µm in size. In contrast, in drinking water samples, the emphasis shifts to smaller particles, with 53% of publications having a detection limit for MPs below 10 µm. For studies analysing human samples, the majority (59%) set the limit of detection below 10 µm, whereas none of the studies have a detection limit above 100 µm, unlike environmental research. This analysis clearly shows how data on MP types may be biased by the chosen analytical method and the detection limit of MP size. 

The analysis of reported data on MP contamination in various food sources and human samples has provided evidence that further research with standardised analysis and methodologies is required. The disparity of protocols for MP extraction and characterisation significantly influences the results, particularly concerning the minimum size of MPs analysed. The predominance of larger MPs in seafood samples contrasts with the focus on smaller particles in drinking water and human studies, reflecting potential health concerns associated with nanoplastic ingestion. 

As the complexities of MP contamination across diverse environmental and biological contexts continues to be investigated, it is evident that a unified approach is essential. This requires the following: (i) the development and universal adoption of standardised protocols for MP identification and characterisation, and (ii) the implementation of methods for the chemical characterisation of MPs in the smaller size range below 10 µm.

#### 3.1.3. Human Exposure to MPs: Particle Shape

To facilitate a comprehensive analysis of the effects of MP morphology on human health, the particles were categorised into five distinct shapes: fragments, fibres, films, pellets, and foams. Various descriptors, as outlined in Methods (Table 2), were employed to classify MPs into these groups. Certain studies within the dataset utilised very specific criteria, such as the aspect ratio, to differentiate between fibrous shapes and fragments. In this context, fibres were characterised as MPs exhibiting a height to width ratio exceeding 3, while particles with an aspect ratio below 1.4 were considered spherical [69,76]. However, the predominant approach among most studies involved visual assessment to discern MPs’ shapes. Accordingly, fibres resembled textile threads, films manifested as thin, two-dimensional particles, pellets were identified as spherical and uniform, foams exhibited high porosity, while fragments appeared irregular and non-uniform in shape.

**Figure 5:**
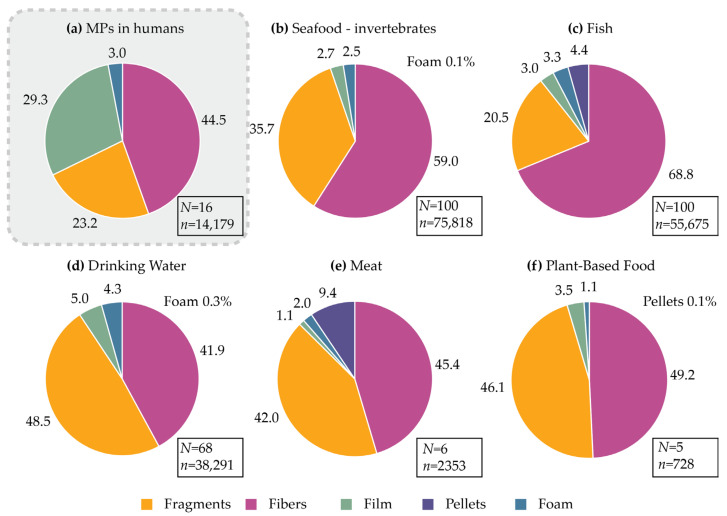
Ratio of five most common microplastic shapes found in different food sources; number represents the percentage drawn from *n*; *N* = number of studies included in the analysis; *n*—total number of microplastics identified.

The distribution of MP shapes in human samples and food sources, obtained from the analysed data, is summarised in Figure 5. Fibres emerge as the most prevalent MP shape, constituting 44.5% of MPs detected in human samples, with high abundance across all food sources. This MP shape is predominant in seafood invertebrates (59%), fish (68.8%), meat (45.4%), and plant-based foods (49.2%), and is the second most common shape in drinking water (41.9%) after fragments. These findings suggest a significant environmental contamination originating from the textile industry, a notion supported by the prevalence of PES, nylon (PA), and PU—commonly utilised textile materials—among the most frequently encountered polymer types in food and human samples (Figure 3). 

The issue of environmental contamination stemming from textile-derived fibres has been extensively reviewed in the literature [68,77,78]. For instance, Napper et al. estimated that a domestic wash load of 6 kg of acrylic fabric could release over 700,000 fibres, with a comparable figure for polyester nearing 500,000 fibres [79]. Notably, the extent of fibre release is heavily dependent upon the garment type rather than solely the material composition. Studies highlighted in the review by Acharya et al. reported that fleece garments exhibit significantly higher microfibre shedding compared to other synthetic materials like blankets or shirts [77]. Consequently, water resources may become contaminated through the discharge of microfibres during laundry processes and inadequate filtration in water treatment facilities. Despite the typically high removal efficiency of drinking water treatment plants, often exceeding 95% [80,81,82], the pervasive contamination of all food sources with fibres suggests that current mitigation measures fall short of effectively averting human exposure.

The data collated from the literature suggest that the second most common MP shape found in human samples is film, which is surprising since this shape represents less than 5% of the MPs found in various food sources. It is important to note that this finding is heavily influenced by the work reported by Yan et al. They found a high proportion of sheet MPs in the stool samples of healthy individuals (49.5%) and patients with inflammatory bowel disease (46.8%) [40]. The analysis of the chemical composition of the sheets found a high prevalence of PET, suggesting food packaging as the main source. The large number of particles reported (8529 occurrences of MPs in total) significantly influenced the analysis, and further work would be required to confirm these findings. Indeed, a number of other studies in humans did not show such a high percentage of films/sheets [41,43,44]. This area of research presents two main challenges: on one hand, there is still a low number of studies involving the evaluation of MPs in human samples; on the other hand, the identification of the MP shape relies on visual assessment, resulting in potential biases. 

The analysis of different MP shapes found in both food and human samples allowed us to draw two key conclusions: firstly, there is a notable prevalence of human exposure to fibres and films; secondly, while regularly shaped spherical particles (pellets) are infrequent, irregularly shaped fragments are highly abundant. 

**Figure 6:**
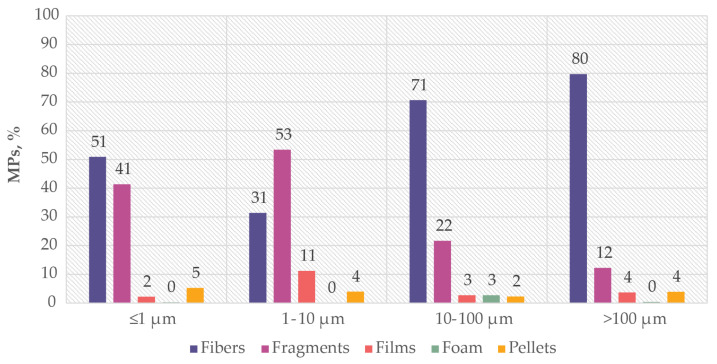
The percentage of occurrence of different shapes of MPs in publications where the minimum size of analysed MPs belongs to the group ≤1 µm, 1–10 µm, 10–100 µm, >100 µm.

The results related to the MP shape analysis can be subject to bias depending on the size limit of the particles examined. Notably, certain shapes such as fibres and foams may be more easily detectable when larger MPs are analysed due to their elongated size and larger volume. To evaluate this aspect better, the publications in our dataset were divided into four groups based on the minimum size of MPs: ≤1 μm, 1–10 μm, 10–100 μm, and >100 μm. The ratio of occurrence of each MP shape across these size groups is presented in Figure 6. 

Notably the occurrence of formats such as foam is reported only in a small number of cases and always in the context of larger particle sizes (>10 µm) potentially due to the challenge of differentiating porosity in small MPs, where this format of material may be described as a fragment.

The average size of MPs detected in different studies varies significantly due to the disparities in size detection limits across different methodologies. Therefore, describing an average size of MPs becomes impractical until standardised methods of size analysis are established. Table 6 summarises the number of studies (*N*) and the number of MPs characterised (*n*) in publications with different size ranges for the smallest analysed MPs. In total, 231 publications analysed MPs in the larger size range (>10 µm), whereas only 101 detected MPs < 10 µm. Interestingly, the number of MPs recorded per study significantly increases when the focus shifts towards smaller sizes (<10 µm). Specifically, an average of 3031 MPs are recorded per study when the size detection limit is ≤1 µm, compared to only 211 MPs per study when the size detection limit is above 100 µm. 

This discrepancy has notable implications, particularly when estimating human exposure to MPs. For instance, a recent study by Qian et al., published in January 2024, analysed MPs in bottled water down to the nanometre scale, estimating the number of MPs at 2.4 ± 1.3 × 10^5^ particles per litre, with the majority being nanoplastics [69]. They suggested that around 90% of the population of plastic particles are not recorded in other reports due to the use of quantification methodologies with lower resolution. Indeed, their estimation of MP concentration is in an order of magnitude higher than in other studies. In the research by Cox et al., published in 2019, the annual MP consumption was estimated between 74,000 and 121,000 [27]. In the dataset utilised for consumption calculations, the highest recorded number of MPs in drinking water was 325 MPs/L (with a size detection limit of MPs 6.5 µm). In a systematic literature review published in 2020, Danopoulos et al. analysed the contamination of drinking water and noted that the maximum reported contamination in bottled water was 4889 MPs/L (with a minimum size of 1 µm) [83,84].

Overall, the data suggest that the number of MPs may increase when methods with higher resolution are employed and when the research focusses on the smaller MP size ranges. Consequently, further investigation into the mean/average size of MPs and the number of MPs to which humans are exposed is necessary with standardised study designs.

### 3.2. Outlook on the Impact of MPs’ Physico-Chemical Properties on Toxicology 

The toxicological effects of MPs have been analysed in various studies, which have highlighted their potential to induce inflammation, apoptosis, cellular DNA damage, and to promote the formation of reactive oxygen species [19,85,86,87]. However, the majority of these reports use the generic term “microplastics” without delving into the specific physico-chemical properties of the fragments, which may influence their interactions with biomolecules. This is an area of great significance that has yet to generate sufficient data to allow a systematic review; however, it is important to present some of the current findings to identify research gaps. In this section, studies where polymer chemistry and particle shape and size have been systematically analysed are discussed, to evaluate how physico-chemical properties can affect the toxicological outcomes associated with MPs exposure.

#### 3.2.1. Toxicology of MPs: Effect of Polymer Chemistry

Research focusing on the toxicity of different polymer types of MPs is scarce, as was highlighted by Thornton Hampton et al., and more than 82% of studies are concentrated on the toxicological effects of PS [65]. Contrary to this, our systematic review revealed that PS accounts for only 2.2% of the most common MP plastic materials found in humans. Therefore, it is imperative to analyse other polymer types as well.

The chemistry of the polymer generating MPs can influence their tissue penetration, cellular uptake, and subsequently their toxicology. For instance, in the study conducted by Stock et al., the transport of MPs of similar-size material through the membrane of Caco cells followed the order PP > PVC > PE, PET, demonstrating that chemical structure significantly impacts the behaviour of MPs within the body [88]. In another study by Ma et al., it was shown that PET and PVC were more toxic to HepG2 cells than PS of similar size and surface charge, inducing oxidative stress and apoptosis in a dose-dependent manner [19]. Similarly, da Silva Brito et al. demonstrated that the cell viability of A549, HEK293, and HeLa cell lines was lower when exposed to PS 1000 nm particles compared to polymethyl methacrylate (PMMA) particles of similar size [89]. Overall, these studies indicate that polymer chemistry can significantly affect cellular response, toxicity, and cellular uptake. 

However, it is not only the polymer chemistry that affects toxicity but also subsequent surface modifications. For example, several studies have shown that the aging of nanoplastics significantly alters their toxicological behaviour. Du et al. demonstrated that artificial aging of PS with UV irradiation changes protein corona formation and uptake by macrophages J774A.1 compared to pristine PS [30], while Wen et al. reported the effects of aging on characteristics of PS, including changes in protein corona, cellular internalisation, and cytotoxicity [31].

Interestingly, Ramsperger et al. showed differences in cellular responses to “supposedly identical” PS MPs from different suppliers [90]. They observed differences in surface charge, residual monomer content, and surface roughness, suggesting that MPs with a more negative ζ-potential and higher monomer content induced a significant metabolic response and altered cell proliferation.

Overall, these studies highlight that not only the polymer type but also subtle changes in surface charge and roughness—such as those induced by environmental weathering—can affect the toxicological effects of MPs. These findings imply the need for more thorough investigations of the toxicological effects of MPs, with detailed characterisations of material properties, in order to draw reliable conclusions.

#### 3.2.2. Toxicology of MPs: Effect of MP Shape

Although the majority of studies on the toxicity and behaviour of MPs in biological systems focusses on spherical particles, as emphasised in several reviews [64,65], it is crucial to recognise that other shapes such as fibres, films, and fragments possess higher surface-to-volume ratios. This characteristic can significantly alter their interactions within biological systems. The importance of particle shape in dictating in vivo behaviour has been explored in the field of drug delivery systems. For instance, the review by A. B. Jindal reported how shape influences the cell uptake, biodistribution, and immune response of nanoparticles [91]. While insights from the field of drug delivery provide valuable preliminary information, comprehensive investigations into the impacts of MP shape on tissue penetration are imperative. To the best of our knowledge, no studies so far have comprehensively analysed the effect of MP shape on tissue penetration and behaviour in biological systems. Understanding how different MP shapes serve as vectors for bacteria, viruses, and pollutants is paramount for elucidating their ecological and health implications. Addressing these research gaps is essential for formulating effective strategies to mitigate the pervasive effects of MPs on both environmental and human health. 

#### 3.2.3. Toxicology of MPs: Effect of MPs’ Size

Generally, researchers agree that smaller MPs possess potentially higher toxicity due to higher tissue penetration and cellular uptake [85,86]. For example, in the study conducted by Zhang et al., it was demonstrated that the uptake of nanosized PS particles (100 and 500 nm) was greater than that of micro-sized particles (1 and 5 µm) in colonic and small intestinal epithelial cells [92]. 

However, the hypothesis that smaller particles may lead to higher cellular uptake may not always be straightforward. Thus, in the research by Stock et al., a higher uptake was observed for PS particles, in the order of particle sizes 4 µm > 1 µm > 10 µm in human intestinal epithelial cells (Caco-2) [93]. They proposed that the increased uptake of 4 µm particles could be attributed to the involvement of two mechanisms: phagocytosis and pinocytosis. In contrast, smaller particles are exclusively absorbed via phagocytosis, while 10 µm MPs exhibited negligible cellular uptake. 

The overall trend of smaller MPs leading to increased tissue penetration was also evidenced in vivo in mice, affecting organs such as the liver, kidney, gut [94], but also the cerebrum, cerebellum, and testis [20]. Consequently, due to their enhanced tissue penetration, smaller MPs may pose a greater threat to human and animal health. For example, in the study by Wang et al., a significantly higher toxicity of 1–10 μm PS MPs was observed compared to 50–100 μm MPs in mice and in vitro in a liver fibrotic injury model [95].

Overall, while the size of MPs is a parameter that has been often evaluated in relation to their toxicological effects, most times this is conducted in isolation from other important physico-chemical characteristics. Most research focuses on spherical particles and uses diameter as the primary metric to characterise size. However, for a comprehensive understanding of the relationship between the morphology of MPs and their toxicological effects, it is crucial to consider size in conjunction with other factors such as shape—especially for fibres—and surface roughness. These additional parameters can reveal more subtle and complex interactions that influence the behaviour and impact of MPs on biological systems. Therefore, future studies should adopt a more holistic approach in analysing the physico-chemical properties of MPs to make more reliable and thorough conclusions about their potential health risks.

## 4. Conclusions

This study has focused on the evaluation of the relationship between the molecular aspects of MPs and their impact on environmental pollution and human health. Through a systematic literature review, the polymer type, shape, and minimum size of MPs across 396 publications encompassing various food sources (including seafood, meat, and plant-based foods), drinking water, and human samples were analysed.

Our results identified the prevalent polymer types found in human samples, which are PES, PA, PU, PP, and PAC, primarily originating from food sources and drinking water. 

Fibres were identified as the most common shape in both human samples and food sources, which can be explained by the widespread use of polymers in textiles and the contamination of food and human exposure. This highlights a potentially overlooked risk associated with the textile industry. Furthermore, our analysis of the size detection limit of MPs in various studies highlighted how the choice of cut-off size can significantly influence the representation of MP shapes and abundance. This emphasises the importance of standardised methodologies in research and underscores the need to focus on smaller MPs for a more comprehensive understanding of their impact.

This review has also highlighted some current limitations in literature, such as the lack of standardised protocols, and lack of data on specific food groups, such as meat and plant-based foods. Addressing these limitations will be crucial for advancing our understanding of the impact of MPs on human health and the environment.

The findings of this study hold potential implications for future evaluations of the toxicological effects of MPs on human health. When designing modelling toxicological studies, researchers should consider shifting their focus towards more abundant materials, rather than solely relying on the commonly used spherical PS [65]. Additionally, the insights of this study can inform the development of strategies to mitigate plastic pollution.

## Figures and Tables

**Table 1:** Search terms used to identify evidence of the presence of MPs in food sources and human samples.

Group	Abbreviation	Search Term 1	Search Terms 2–3
Seafood: invertebrates	I	microplastic *	2) digestive OR gut OR ingestion OR uptake 3) Mussel OR oyster OR clam OR lobster OR shrimp OR sea urchin OR snail OR sea cucumbers OR crab
Fish	F		2) digestive OR gut OR ingestion OR uptake 3) fish
Drinking Water	DW		2) drinking water
Meat	M		2) meat OR pork OR beef OR chicken OR goat OR turkey OR duck OR lamb OR buffalo
Plant-Based food	P		2) edible plant OR vegetable OR fruit OR herb OR plant based
Humans	H		2) digestive OR gut OR ingestion OR uptake OR internalization OR tissue OR organ OR bioaccumulation 3) human *

**Table 2:** Terms included into the description of MPs shapes used in this study.

Shape Group	Terms Included
Fibres	Fibre, filament, thread, line, fibrous MP
Fragments	Fragment, angular, irregularly shaped
Films	Film, sheet, flat MP
Foams	Foam, sponge-like
Pellets	Pellet, bead, sphere

**Table 3:** Food groups and type of food mentioned in the dataset.

Food Group	Food Sources Included in the Database	*N* Studies
Seafood Invertebrates	Mussels, oysters, clams, lobsters, shrimps, sea urchins, snails, sea cucumbers, crabs	139
Fish	Freshwater and seawater fish, wild (only edible species) or farmed, canned fish, fishmeal	135
Drinking Water	Tap water, bottled water, borehole drinking water, water after purification in drinking water treatment plants	83
Meat	chicken, beef, pork, chicken eggs, duck (edible species)	8
Plant-Based food	Edible fruits and vegetables, lettuce, vegetable oil, processed plant-based food	5

**Table 4:** A summary of studies used for the analysis of MP occurrence in humans; *N*—number of participants in the study; *n*—number of particles confirmed as microplastics (MPs); Size_min_—minimum size of MPs analysed; Ref.—reference; NM—data not mentioned.

Health Status	Sample Analysed	*N* Patients	*n* MPs	Size_min_	Ref.
**Gastrointestinal Tract**					
healthy	stool	8	727	50 μm	[39]
healthy and inflammatory bowel disease	stool	102	8529	5 μm	[40]
healthy and liver cirrhosis	liver tissue	6	102	4 μm	[41]
colorectal cancer and normal colon	colon tissue	11	3638	NM	[42]
healthy	stool	8	129	30 μm	[43]
healthy	urine and kidney tissue	10	26	1 μm	[44]
healthy	stool	26	213	20 μm	[45]
healthy, colorectal adenocarcinoma	colon tissues	32	3	1 μm	[46]
various conditions	lung, intestine, tonsil tissues	41	37	20 μm	[47]
**Vascular system/body fluids**					
thrombosis	thrombi	26	1	2.1 μm	[48]
patients undergoing surgery	saphenous vein tissue	5	102	5 μm	[49]
NM	blood, cerebrospinal fluid, effusions and cyst fluids	104	17	2 μm	[50]
patients undergoing cardiac surgery	blood, vascular tissues	15	17,961	20 μm	[51]
healthy	blood	22	27	0.7 μm	[52]
atherosclerosis	coronary artery, carotid artery, carotid aortic samples	17	628	NM	[53]
**Reproductive system and new-borns health**					
healthy	breastmilk	34	41	2 μm	[54]
healthy infants	meconium	37	0	10 μm	[55]
preterm birth	amniotic fluid and placenta	20	21	10 μm	[56]
healthy	semen	10	16	2 μm	[57]
healthy	placentas and meconium	36	941	20 μm	[58]
healthy	placentas	30	142	1 μm	[59]
healthy, endometriosis	urine	38	355	5 μm	[21]
acute caesarean sections	maternal amniotic fluid	40	776	20 μm	[60]
healthy	testis, semen	30	56	20 μm	[61]
**Other systems**					
hip or knee arthroplasty	lower limb joints	45	343	11 μm	[62]
ocular diseases	vitreous samples	49	1745	20 μm	[63]

**Table 5:** Characteristics and uses of polymer types most commonly identified as microplastics.

Polymer	Global Production, Mt [66,67]	Chemical Structure	Examples of Uses [67]
**PES (textiles)**	63.3	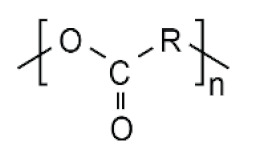	Apparel, home textiles, water filtration systems, conveyor belts, safety belts, geotextiles, non-woven fabrics, medical masks, etc.
**PES (PET)**	27.6		Bottles for water, soft drinks, juices, cleaners, etc., food jars/pots, clothes, plastic films, microwavable packaging, etc.
**PA**	6.1	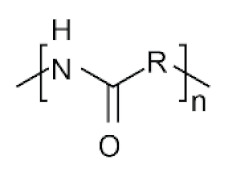	Fibres, bristles for toothbrushes, tubing, clothing, fishing lines and nets, conveyor belts, food packaging, medical devices, etc.
**PU**	28.4	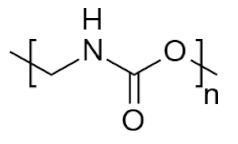	Building insulation, pillows and mattresses, insulating foams, surface coatings, rollers for printing used in cars, carpet, flexible foam in furniture, elastic fibre, etc.
**PP**	69.3	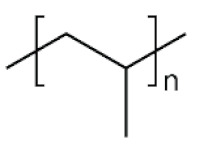	Food packaging, sweets and snacks, wrappers, hinged caps, microwave containers, pipes, automotive parts, bank notes, etc.
**PAC**	-	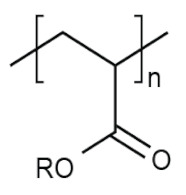	diapers, sanitary napkins, textile finishing, nail polishes, and skincare products, contact lenses, touch screens, paper coatings, etc.
**PE**	106.6	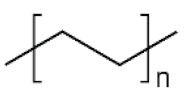	Reusable bags, trays and containers, agricultural film, food packaging film, toys, milk bottles, shampoo bottles, pipes, houseware, etc.
**PVC**	35.9	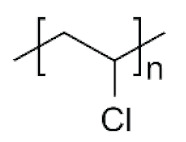	Window frames, pipes, plumbing and guttering, shower curtains, synthetic leather, cosmetic containers, commercial cling wrap, etc.
**PS**	23.0	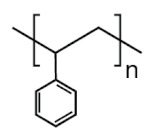	Food packaging (dairy, fishery), electrical and electronic equipment, inner liner for fridges, eyeglasses frames, food containers, disposable cups, plates and cutlery, boxes for compact discs and cassettes, etc.

**Table 6:** Summary of number of studies used for the analyses (*N*) and number of MPs characterised (*n*) grouped according to the size detection limit of MPs.

Size Group	*N* Studies	*n* MPs	MPs/Study
≤1 μm	38	115,183	3031
1–10 μm	63	59,038	937
10–100 μm	147	106,660	726
>100 μm	84	17,696	211

## Supplementary Materials

The following supporting information can be downloaded at: https://www.mdpi.com/article/10.3390/ijms25137074/s1.
