# Supplementary material for: Mapping Microplastics in Humans: Analysis of Polymer Types, and Shapes in Food and Drinking Water—A Systematic Review
Source: Int J Mol Sci. 2024 Jun 27;25(13):7074. doi: 10.3390/ijms25137074 (PMC11241750; doi:10.3390/ijms25137074)
Supplement: Supplementary file 1 [file ijms-25-07074-s001.zip › ijms-3034278-supplementary.pdf]

*Supplementary material*

# Mapping Microplastics in Humans: Analysis of Polymer Types and Shapes in Food and Drinking Water - a Systematic Review

**Alena Vdovchenko [1], Marina Resmini [1,*]**

[1] Department of Chemistry, School of Physical and Chemical Sciences, Queen Mary University of London, London E1 4NS, UK; a.vdovchenko@qmul.ac.uk
* Correspondence: m.resmini@qmul.ac.uk (M.R.)

**Table S1.** List of references used as evidence sources for different food groups and humans.

| **Seafood: Invertebrates** |
|---|
| 1. Ogunola, S. O., Reis-Santos, P., Wootton, N., & Gillanders, B. M. (2022). Microplastics in decapod crustaceans sourced from Australian seafood markets. *Marine Pollution Bulletin*, *179*(113706), 113706. doi:10.1016/j.marpolbul.2022.113706<br>2. Joshy, A., Krupesha Sharma, S. R., & Mini, K. G. (2022). Microplastic contamination in commercially important bivalves from the southwest coast of India. *Environmental Pollution (Barking, Essex: 1987)*, *305*(119250), 119250. doi:10.1016/j.envpol.2022.119250<br>3. Oliveira, A. R., Sardinha-Silva, A., Andrews, P. L. R., Green, D., Cooke, G. M., Hall, S., … Sykes, A. V. (2020). Microplastics presence in cultured and wild-caught cuttlefish, Sepia officinalis. *Marine Pollution Bulletin*, *160*(111553), 111553. doi:10.1016/j.marpolbul.2020.111553<br>4. Hermabessiere, L., Paul-Pont, I., Cassone, A.-L., Himber, C., Receveur, J., Jezequel, R., … Soudant, P. (2019). Microplastic contamination and pollutant levels in mussels and cockles collected along the channel coasts. *Environmental Pollution (Barking, Essex: 1987)*, *250*, 807–819. doi:10.1016/j.envpol.2019.04.051<br>5. Polt, L., Motyl, L., & Fischer, E. K. (2023). Abundance and distribution of microplastics in invertebrate and fish species and sediment samples along the German Wadden Sea coastline. *Animals: An Open Access Journal from MDPI*, *13*(10). doi:10.3390/ani13101698<br>6. Zhang, D., Fraser, M. A., Huang, W., Ge, C., Wang, Y., Zhang, C., & Guo, P. (2021). Microplastic pollution in water, sediment, and specific tissues of crayfish (Procambarus clarkii) within two different breeding modes in Jianli, Hubei province, China. *Environmental Pollution (Barking, Essex: 1987)*, *272*(115939), 115939. doi:10.1016/j.envpol.2020.115939<br>7. Marques, F., Vale, C., Rudnitskaya, A., Moreirinha, C., Costa, S. T., & Botelho, M. J. (2021). Major characteristics of microplastics in mussels from the Portuguese coast. *Environmental Research*, *197*(110993), 110993. doi:10.1016/j.envres.2021.110993<br>8. Doan, T. O., Duong, T. T., Pham, L. A., Nguyen, T. M., Pham, P. T., Hoang, T. Q., … Le, T. P. Q. (2023). Microplastic accumulation in bivalves collected from different coastal areas of Vietnam and an assessment of potential risks. *Environmental Monitoring and Assessment*, *195*(12), 1511. doi:10.1007/s10661-023-12087-8<br>9. Ojeda, M., Cossi, P. F., Rimondino, G. N., Chiesa, I. L., Boy, C. C., & Pérez, A. F. (2021). Microplastics pollution in the intertidal limpet, Nacella magellanica, from Beagle Channel (Argentina). *The Science of the Total Environment*, *795*(148866), 148866. doi:10.1016/j.scitotenv.2021.148866<br>10. Karlsson, T. M., Vethaak, A. D., Almroth, B. C., Ariese, F., van Velzen, M., Hassellöv, M., & Leslie, H. A. (2017). Screening for microplastics in sediment, water, marine invertebrates and fish: Method development and microplastic accumulation. *Marine Pollution Bulletin*, *122*(1–2), 403–408. doi:10.1016/j.marpolbul.2017.06.081<br>11. Hossain, M. S., Rahman, M. S., Uddin, M. N., Sharifuzzaman, S. M., Chowdhury, S. R., Sarker, S., & Nawaz Chowdhury, M. S. (2020). Microplastic contamination in Penaeid shrimp from the Northern Bay of Bengal. *Chemosphere*, *238*(124688), 124688. doi:10.1016/j.chemosphere.2019.124688<br>12. Sevillano-González, M., González-Sálamo, J., Díaz-Peña, F. J., Hernández-Sánchez, C., Catalán Torralbo, S., Ródenas Seguí, A., & Hernández-Borges, J. (2022). Assessment of microplastic content |

in Diadema africanum sea urchin from Tenerife (Canary Islands, Spain). *Marine Pollution Bulletin*, *175*(113174), 113174. doi:10.1016/j.marpolbul.2021.113174

13. Rowenczyk, L., Cai, H., Nguyen, B., Sirois, M., Côté-Laurin, M.-C., Toupoint, N., … Tufenkji, N. (2022). From freshwaters to bivalves: Microplastic distribution along the Saint-Lawrence river-to-sea continuum. *Journal of Hazardous Materials*, *435*(128977), 128977. doi:10.1016/j.jhazmat.2022.128977
14. Covernton, G. A., Cox, K. D., Fleming, W. L., Buirs, B. M., Davies, H. L., Juanes, F., … Dower, J. F. (2022). Large size (>100-μm) microplastics are not biomagnifying in coastal marine food webs of British Columbia, Canada. *Ecological Applications: A Publication of the Ecological Society of America*, *32*(7), e2654. doi:10.1002/eap.2654
15. Rahmawati, Krisanti, M., Riani, E., & Cordova, M. R. (2023). Microplastic contamination in the digestive tract of sea urchins (Echinodermata: Echinoidea) in Kepulauan Seribu, Indonesia. *Environmental Monitoring and Assessment*, *195*(9), 1103. doi:10.1007/s10661-023-11655-2
16. Murano, C., Vaccari, L., Casotti, R., Corsi, I., & Palumbo, A. (2022). Occurrence of microfibres in wild specimens of adult sea urchin Paracentrotus lividus (Lamarck, 1816) from a coastal area of the central Mediterranean Sea. *Marine Pollution Bulletin*, *176*(113448), 113448. doi:10.1016/j.marpolbul.2022.113448
17. Cau, A., Avio, C. G., Dessì, C., Follesa, M. C., Moccia, D., Regoli, F., & Pusceddu, A. (2019). Microplastics in the crustaceans Nephrops norvegicus and Aristeus antennatus: Flagship species for deep-sea environments? *Environmental Pollution (Barking, Essex: 1987)*, *255*(Pt 1), 113107. doi:10.1016/j.envpol.2019.113107
18. Mohsen, M., Wang, Q., Zhang, L., Sun, L., Lin, C., & Yang, H. (2019). Microplastic ingestion by the farmed sea cucumber Apostichopus japonicus in China. *Environmental Pollution (Barking, Essex: 1987)*, *245*, 1071–1078. doi:10.1016/j.envpol.2018.11.083
19. Zhao, S., Ward, J. E., Danley, M., & Mincer, T. J. (2018). Field-based evidence for microplastic in marine aggregates and mussels: Implications for trophic transfer. *Environmental Science & Technology*, *52*(19), 11038–11048. doi:10.1021/acs.est.8b03467
20. Lozano-Hernández, E. A., Ramírez-Álvarez, N., Rios Mendoza, L. M., Macías-Zamora, J. V., Sánchez-Osorio, J. L., & Hernández-Guzmán, F. A. (2021). Microplastic concentrations in cultured oysters in two seasons from two bays of Baja California, Mexico. *Environmental Pollution (Barking, Essex: 1987)*, *290*(118031), 118031. doi:10.1016/j.envpol.2021.118031
21. Raguso, C., Grech, D., Becchi, A., Ubaldi, P. G., Lasagni, M., Guala, I., & Saliu, F. (2022). Detection of microplastics and phthalic acid esters in sea urchins from Sardinia (Western Mediterranean Sea). Marine Pollution Bulletin, 185(Pt A), 114328. doi:10.1016/j.marpolbul.2022.114328
22. Davidson, K., & Dudas, S. E. (2016). Microplastic ingestion by wild and cultured Manila clams (Venerupis philippinarum) from Baynes Sound, British Columbia. Archives of Environmental Contamination and Toxicology, 71(2), 147–156. doi:10.1007/s00244-016-0286-4
23. Atici, A. A. (2022). The first evidence of microplastic uptake in natural freshwater mussel, Unio stevenianus from Karasu River, Turkey. Biomarkers: Biochemical Indicators of Exposure, Response, and Susceptibility to Chemicals, 27(2), 118–126. doi:10.1080/1354750X.2021.2020335
24. Rapp, J., Herrera, A., Bondyale-Juez, D. R., González-Pleiter, M., Reinold, S., Asensio, M., … Gómez, M. (2021). Microplastic ingestion in jellyfish Pelagia noctiluca (Forsskal, 1775) in the North Atlantic Ocean. Marine Pollution Bulletin, 166(112266), 112266. doi:10.1016/j.marpolbul.2021.112266
25. Mohsen, M., Lin, C., Liu, S., & Yang, H. (2022). Existence of microplastics in the edible part of the sea cucumber Apostichopus japonicus. Chemosphere, 287(Pt 1), 132062. doi:10.1016/j.chemosphere.2021.132062
26. Wang, D., Su, L., Ruan, H. D., Chen, J., Lu, J., Lee, C.-H., & Jiang, S. Y. (2021). Quantitative and qualitative determination of microplastics in oyster, seawater and sediment from the coastal areas in Zhuhai, China. Marine Pollution Bulletin, 164(112000), 112000. doi:10.1016/j.marpolbul.2021.112000
27. Lombardo, J., Solomando, A., Cohen-Sánchez, A., Pinya, S., Tejada, S., Ferriol, P., … Sureda, A. (2022). Effects of human activity on markers of oxidative stress in the intestine of Holothuria tubulosa, with special reference to the presence of microplastics. International Journal of Molecular Sciences, 23(16), 9018. doi:10.3390/ijms23169018
28. Guo, Q., Ding, C., Li, Z., Chen, X., Wu, J., Li, X., … Chen, T. (2023). Characteristics and potential human health risks of microplastics identified in typical clams from South Yellow Sea Mudflat. The Science of the Total Environment, 905(167044), 167044. doi:10.1016/j.scitotenv.2023.167044

29. Pagter, E., Nash, R., Frias, J., & Kavanagh, F. (2021). Assessing microplastic distribution within infaunal benthic communities in a coastal embayment. The Science of the Total Environment, 791(148278), 148278. doi:10.1016/j.scitotenv.2021.148278
30. Gedik, K., & Eryaşar, A. R. (2020). Microplastic pollution profile of Mediterranean mussels (Mytilus galloprovincialis) collected along the Turkish coasts. Chemosphere, 260(127570), 127570. doi:10.1016/j.chemosphere.2020.127570
31. Schuab, J. M., de Paula, M. S., Ocaris, E. R. Y., Milagres, M. R., Motta, D. G., & da Costa, M. B. (2023). First record of microplastic in the Brazilian sea hare Aplysia brasiliana Rang, 1828 (Mollusca: Aplysiidae). *The Science of the Total Environment*, *895*(165156), 165156. doi:10.1016/j.scitotenv.2023.165156
32. Kieu-Le, T.-C., Tran, Q.-V., Truong, T.-N.-S., & Strady, E. (2022). Anthropogenic fibres in white clams, Meretrix lyrata, cultivated downstream a developing megacity, Ho Chi Minh City, Viet Nam. *Marine Pollution Bulletin*, *174*(113302), 113302. doi:10.1016/j.marpolbul.2021.113302
33. Muhammad Husin, M. J., Mazlan, N., Shalom, J., Saud, S. N., & Abdullah Sani, M. S. (2021). Evaluation of microplastics ingested by sea cucumber Stichopus horrens in Pulau Pangkor, Perak, Malaysia. *Environmental Science and Pollution Research International*, *28*(43), 61592–61600. doi:10.1007/s11356-021-15099-4
34. Sparks, C., Awe, A., & Maneveld, J. (2021). Abundance and characteristics of microplastics in retail mussels from Cape Town, South Africa. *Marine Pollution Bulletin*, *166*(112186), 112186. doi:10.1016/j.marpolbul.2021.112186
35. Jitkaew, P., Pradit, S., Noppradit, P., Sengloyluan, K., Yucharoen, M., Suwanno, S., … Nitiratsuwan, T. (2023). Occurrence of microplastics in freshwater gastropods from a tropical river U-Taphao, southern Thailand. *PeerJ*, *11*, e14861. doi:10.7717/peerj.14861
36. Carreras-Colom, E., Constenla, M., Soler-Membrives, A., Cartes, J. E., Baeza, M., Padrós, F., & Carrassón, M. (2018). Spatial occurrence and effects of microplastic ingestion on the deep-water shrimp Aristeus antennatus. *Marine Pollution Bulletin*, *133*, 44–52. doi:10.1016/j.marpolbul.2018.05.012
37. Phuong, N. N., Zalouk-Vergnoux, A., Kamari, A., Mouneyrac, C., Amiard, F., Poirier, L., & Lagarde, F. (2018). Quantification and characterization of microplastics in blue mussels (Mytilus edulis): protocol setup and preliminary data on the contamination of the French Atlantic coast. *Environmental Science and Pollution Research International*, *25*(7), 6135–6144. doi:10.1007/s11356-017-8862-3
38. Keshavarzifard, M., Vazirzadeh, A., & Sharifinia, M. (2021). Occurrence and characterization of microplastics in white shrimp, Metapenaeus affinis, living in a habitat highly affected by anthropogenic pressures, northwest Persian Gulf. *Marine Pollution Bulletin*, *169*(112581), 112581. doi:10.1016/j.marpolbul.2021.112581
39. Yu, F., Pei, Y., Zhang, X., Wu, X., Zhang, G., & Ma, J. (2023). Occurrence and distribution characteristics of aged microplastics in the surface water, sediment, and crabs of the aquaculture pond in the Yangtze River Delta of China. *The Science of the Total Environment*, *871*(162039), 162039. doi:10.1016/j.scitotenv.2023.162039
40. Abbasi, S., Soltani, N., Keshavarzi, B., Moore, F., Turner, A., & Hassanaghaei, M. (2018). Microplastics in different tissues of fish and prawn from the Musa Estuary, Persian Gulf. *Chemosphere*, *205*, 80–87. doi:10.1016/j.chemosphere.2018.04.076
41. Vinay Kumar, B. N., Löschel, L. A., Imhof, H. K., Löder, M. G. J., & Laforsch, C. (2021). Analysis of microplastics of a broad size range in commercially important mussels by combining FTIR and Raman spectroscopy approaches. *Environmental Pollution (Barking, Essex: 1987)*, *269*(116147), 116147. doi:10.1016/j.envpol.2020.116147
42. Zhu, W., Liu, W., Chen, Y., Liao, K., Yu, W., & Jin, H. (2023). Microplastics in Antarctic krill (Euphausia superba) from Antarctic region. *The Science of the Total Environment*, *870*(161880), 161880. doi:10.1016/j.scitotenv.2023.161880
43. Mayoma, B. S., Sørensen, C., Shashoua, Y., & Khan, F. R. (2020). Microplastics in beach sediments and cockles (Anadara antiquata) along the Tanzanian coastline. *Bulletin of Environmental Contamination and Toxicology*, *105*(4), 513–521. doi:10.1007/s00128-020-02991-x
44. Hara, J., Frias, J., & Nash, R. (2020). Quantification of microplastic ingestion by the decapod crustacean Nephrops norvegicus from Irish waters. *Marine Pollution Bulletin*, *152*(110905), 110905. doi:10.1016/j.marpolbul.2020.110905
45. Chen, C.-F., Ju, Y.-R., Lim, Y. C., Wang, M.-H., Chen, C.-W., & Dong, C.-D. (2023). Microplastics in coastal farmed oyster (Crassostrea angulata) shells: Abundance, characteristics, and diversity. *Marine Pollution Bulletin*, *194*(Pt A), 115228. doi:10.1016/j.marpolbul.2023.115228

46. Abelouah, M. R., Romdhani, I., Ben-Haddad, M., Hajji, S., De-la-Torre, G. E., Gaaied, S., … Ait Alla, A. (2023). Binational survey using Mytilus galloprovincialis as a bioindicator of microplastic pollution: Insights into chemical analysis and potential risk on humans. *The Science of the Total Environment*, *870*(161894), 161894. doi:10.1016/j.scitotenv.2023.161894
47. Soltani, N., Amini-Birami, F., Keshavarzi, B., Moore, F., Busquets, R., Sorooshian, A., … Shahraki, A. R. (2023). Microplastic occurrence in selected aquatic species of the Persian Gulf: No evidence of trophic transfer or effect of diet. *The Science of the Total Environment*, *892*(164685), 164685. doi:10.1016/j.scitotenv.2023.164685
48. Mladinich, K., Holohan, B. A., Shumway, S. E., & Ward, J. E. (2023). The relationship between microplastics in eastern oysters (Crassostrea virginica) and surrounding environmental compartments in Long Island Sound. *Marine Environmental Research*, *189*(106040), 106040. doi:10.1016/j.marenvres.2023.106040
49. Yozukmaz, A. (2021). Investigation of microplastics in edible wild mussels from İzmir Bay (Aegean Sea, Western Turkey): A risk assessment for the consumers. *Marine Pollution Bulletin*, *171*(112733), 112733. doi:10.1016/j.marpolbul.2021.112733
50. Railo, S., Talvitie, J., Setälä, O., Koistinen, A., & Lehtiniemi, M. (2018). Application of an enzyme digestion method reveals microlitter in Mytilus trossulus at a wastewater discharge area. *Marine Pollution Bulletin*, *130*, 206–214. doi:10.1016/j.marpolbul.2018.03.022
51. Ding, J., Li, J., Sun, C., Jiang, F., He, C., Zhang, M., … Ding, N. X. (2020). An examination of the occurrence and potential risks of microplastics across various shellfish. *The Science of the Total Environment*, *739*(139887), 139887. doi:10.1016/j.scitotenv.2020.139887
52. Ding, J., Sun, C., He, C., Li, J., Ju, P., & Li, F. (2021). Microplastics in four bivalve species and basis for using bivalves as bioindicators of microplastic pollution. *The Science of the Total Environment*, *782*(146830), 146830. doi:10.1016/j.scitotenv.2021.146830
53. Chen, J.-C., Fang, C., Zheng, R.-H., Hong, F.-K., Jiang, Y.-L., Zhang, M., … Lin, L.-S. (2021). Microplastic pollution in wild commercial nekton from the South China Sea and Indian Ocean, and its implication to human health. *Marine Environmental Research*, *167*(105295), 105295. doi:10.1016/j.marenvres.2021.105295
54. Saha, M., Naik, A., Desai, A., Nanajkar, M., Rathore, C., Kumar, M., & Gupta, P. (2021). Microplastics in seafood as an emerging threat to marine environment: A case study in Goa, west coast of India. *Chemosphere*, *270*(129359), 129359. doi:10.1016/j.chemosphere.2020.129359
55. Leung, M. M.-L., Ho, Y.-W., Lee, C.-H., Wang, Y., Hu, M., Kwok, K. W. H., … Fang, J. K.-H. (2021). Improved Raman spectroscopy-based approach to assess microplastics in seafood. *Environmental Pollution (Barking, Essex: 1987)*, *289*(117648), 117648. doi:10.1016/j.envpol.2021.117648
56. Tee-Hor, K., Nitiratsuwan, T., & Pradit, S. (2023). Identification of anthropogenic debris in the stomach and intestines of giant freshwater prawns from the Trang River in southern Thailand. *PeerJ*, *11*, e16082. doi:10.7717/peerj.16082
57. Mazlan, N., Shukhairi, S. S., Muhammad Husin, M. J., Shalom, J., Saud, S. N., Abdullah Sani, M. S., … Sopian, N. A. (2023). Evaluation of microplastics isolated from sea cucumber Acaudina molpadioides in Pulau Langkawi, Malaysia. *Heliyon*, *9*(6), e16822. doi:10.1016/j.heliyon.2023.e16822
58. Zhao, S., Liu, Y., Sun, C., Wang, X., Hou, C., Teng, J., … Wang, Q. (2023). The pollution characteristics and risk assessment of microplastics in mollusks collected from the Bohai Sea. *The Science of the Total Environment*, 169739. doi:10.1016/j.scitotenv.2023.169739
59. Aung, T., Batish, I., & Ovissipour, R. (2022). Prevalence of microplastics in the Eastern oyster Crassostrea virginica in the Chesapeake Bay: The impact of different digestion methods on microplastic properties. *Toxics*, *10*(1), 29. doi:10.3390/toxics10010029
60. Digka, N., Tsangaris, C., Torre, M., Anastasopoulou, A., & Zeri, C. (2018). Microplastics in mussels and fish from the Northern Ionian Sea. *Marine Pollution Bulletin*, *135*, 30–40. doi:10.1016/j.marpolbul.2018.06.063
61. Başaran Kankılıç, G., Koraltan, İ., Erkmen, B., Çağan, A. S., Çırak, T., Özen, M., … Tavşanoğlu, Ü. N. (2023). Size-selective microplastic uptake by freshwater organisms: Fish, mussel, and zooplankton. *Environmental Pollution (Barking, Essex: 1987)*, *336*(122445), 122445. doi:10.1016/j.envpol.2023.122445
62. Li, Z., Chao, M., He, X., Lan, X., Tian, C., Feng, C., & Shen, Z. (2022). Microplastic bioaccumulation in estuary-caught fishery resource. *Environmental Pollution (Barking, Essex: 1987)*, *306*(119392), 119392. doi:10.1016/j.envpol.2022.119392
63. Piarulli, S., Scapinello, S., Sciutto, G., Prati, S., Mazzeo, R., Booth, A. M., & Airoldi, L. (2022). Quantifying spatial variation in the uptake of microplastic by mussels using biodeposit traps: A

field-based study. *Marine Pollution Bulletin*, *174*(113305), 113305. doi:10.1016/j.marpolbul.2021.113305
64. Digka, N., Patsiou, D., Kaberi, H., Krasakopoulou, E., & Tsangaris, C. (2023). Microplastic ingestion and its effects on sea urchin Paracentrotus lividus: A field study in a coastal East Mediterranean environment. *Marine Pollution Bulletin*, *196*(115613), 115613. doi:10.1016/j.marpolbul.2023.115613
65. Qu, X., Su, L., Li, H., Liang, M., & Shi, H. (2018). Assessing the relationship between the abundance and properties of microplastics in water and in mussels. *The Science of the Total Environment*, *621*, 679–686. doi:10.1016/j.scitotenv.2017.11.284
66. Klasios, N., De Frond, H., Miller, E., Sedlak, M., & Rochman, C. M. (2021). Microplastics and other anthropogenic particles are prevalent in mussels from San Francisco Bay, and show no correlation with PAHs. *Environmental Pollution (Barking, Essex: 1987)*, *271*(116260), 116260. doi:10.1016/j.envpol.2020.116260
67. Bošković, N., Joksimović, D., & Bajt, O. (2023). Microplastics in mussels from the Boka Kotorska Bay (Adriatic Sea) and impact on human health. *Food and Chemical Toxicology: An International Journal Published for the British Industrial Biological Research Association*, *173*(113641), 113641. doi:10.1016/j.fct.2023.113641
68. Zhu, J., Zhang, Q., Huang, Y., Jiang, Y., Li, J., Michal, J. J., … Lan, W. (2021). Long-term trends of microplastics in seawater and farmed oysters in the Maowei Sea, China. *Environmental Pollution (Barking, Essex: 1987)*, *273*(116450), 116450. doi:10.1016/j.envpol.2021.116450
69. Ehlers, S. M., Ellrich, J. A., & Koop, J. H. E. (2022). Microplastic load and polymer type composition in European rocky intertidal snails: Consistency across locations, wave exposure and years. *Environmental Pollution (Barking, Essex: 1987)*, *292*(Pt A), 118280. doi:10.1016/j.envpol.2021.118280
70. Du, Y., Zhao, J., Teng, J., Ren, J., Zheng, P., Zhu, X., … Wang, Q. (2022). Seasonal change of microplastics uptake in the Pacific oysters Crassostrea gigas cultured in the Yellow Sea and Bohai Sea, China. *Marine Pollution Bulletin*, *185*(Pt B), 114341. doi:10.1016/j.marpolbul.2022.114341
71. Aminah, I. S., Ikejima, K., & Vermeiren, P. (2023). Ingestion and translocation of microplastics in tissues of deposit-feeding crabs (Grapsoidea, Ocypodoidea) in Kochi estuary, Japan. *Marine Environmental Research*, *192*(106252), 106252. doi:10.1016/j.marenvres.2023.106252
72. Gérigny, O., Pedrotti, M.-L., El Rakwe, M., Brun, M., Pavec, M., Henry, M., … Galgani, F. (2022). Characterization of floating microplastic contamination in the bay of Marseille (French Mediterranean Sea) and its impact on zooplankton and mussels. *Marine Pollution Bulletin*, *175*(113353), 113353. doi:10.1016/j.marpolbul.2022.113353
73. Ribeiro, V. V., Avelino Soares, T. M., De-la-torre, G. E., Casado-Coy, N., Sanz-Lazaro, C., & Castro, Í. B. (2024). Microplastics in rocky shore mollusks of different feeding habits: An assessment of sentinel performance. *Environmental Pollution (Barking, Essex: 1987)*, *346*(123571), 123571. doi:10.1016/j.envpol.2024.123571
74. Wu, Y., Yang, J., Li, Z., He, H., Wang, Y., Wu, H., … Wang, L. (2022). How does bivalve size influence microplastics accumulation? *Environmental Research*, *214*(Pt 1), 113847. doi:10.1016/j.envres.2022.113847
75. Plee, T. A., & Pomory, C. M. (2020). Microplastics in sandy environments in the Florida Keys and the panhandle of Florida, and the ingestion by sea cucumbers (Echinodermata: Holothuroidea) and sand dollars (Echinodermata: Echinoidea). *Marine Pollution Bulletin*, *158*(111437), 111437. doi:10.1016/j.marpolbul.2020.111437
76. Kazour, M., & Amara, R. (2020). Is blue mussel caging an efficient method for monitoring environmental microplastics pollution? *The Science of the Total Environment*, *710*(135649), 135649. doi:10.1016/j.scitotenv.2019.135649
77. Quaglia, N. C., Capuozzo, F., Ceci, E., Cometa, S., Di Pinto, A., Mottola, A., … Dambrosio, A. (2023). Preliminary survey on the occurrence of microplastics in bivalve mollusks marketed in Apulian fish markets. *Italian Journal of Food Safety*, *12*(2), 10906. doi:10.4081/ijfs.2023.10906
78. Carreras-Colom, E., Constenla, M., Soler-Membrives, A., Cartes, J. E., Baeza, M., & Carrassón, M. (2020). A closer look at anthropogenic fiber ingestion in Aristeus antennatus in the NW Mediterranean Sea: Differences among years and locations and impact on health condition. *Environmental Pollution (Barking, Essex: 1987)*, *263*(Pt A), 114567. doi:10.1016/j.envpol.2020.114567
79. Leila, B., Sedláček, P., & Anastasopoulou, A. (2023). Plastic pollution in the deep-sea Giant red shrimp, Aristaeomorpha foliacea, in the Eastern Ionian Sea; an alarm point on stock and human health safety. *The Science of the Total Environment*, *877*(162783), 162783. doi:10.1016/j.scitotenv.2023.162783

80. Dowarah, K., Patchaiyappan, A., Thirunavukkarasu, C., Jayakumar, S., & Devipriya, S. P. (2020). Quantification of microplastics using Nile Red in two bivalve species Perna viridis and Meretrix meretrix from three estuaries in Pondicherry, India and microplastic uptake by local communities through bivalve diet. *Marine Pollution Bulletin*, *153*(110982), 110982. doi:10.1016/j.marpolbul.2020.110982
81. Minder, M. L., Colombo, I. G., & Rountos, K. J. (2023). Baseline assessment of microplastics in commercially important marine bivalves from New York, U.S.A. *Marine Pollution Bulletin*, *188*(114625), 114625. doi:10.1016/j.marpolbul.2023.114625
82. Can Tunçelli, İ., & Erkan, N. (2023). Microplastic pollution in wild and aquacultured Mediterranean mussels from the Sea of Marmara: Abundance, characteristics, and health risk estimations. *Environmental Research*, 117787. doi:10.1016/j.envres.2023.117787
83. Teng, J., Wang, Q., Ran, W., Wu, D., Liu, Y., Sun, S., … Zhao, J. (2019). Microplastic in cultured oysters from different coastal areas of China. *The Science of the Total Environment*, *653*, 1282–1292. doi:10.1016/j.scitotenv.2018.11.057
84. Otegui, M. B. P., Schuab, J. M., França, M. A., Caniçali, F. B., Yapuchura, E. R., Zamprogno, G. C., & da Costa, M. B. (2024). Microplastic contamination in different shell length in Tivela mactroides (Born, 1778). *The Science of the Total Environment*, *922*(171283), 171283. doi:10.1016/j.scitotenv.2024.171283
85. Vermeiren, P., Ikejima, K., Uchida, Y., & C Muñoz, C. (2023). Microplastic distribution among estuarine sedimentary habitats utilized by intertidal crabs. *The Science of the Total Environment*, *866*(161400), 161400. doi:10.1016/j.scitotenv.2023.161400
86. Feng, Z., Wang, R., Zhang, T., Wang, J., Huang, W., Li, J., … Gao, G. (2020). Microplastics in specific tissues of wild sea urchins along the coastal areas of northern China. *The Science of the Total Environment*, *728*(138660), 138660. doi:10.1016/j.scitotenv.2020.138660
87. Zhang, T., Sun, Y., Song, K., Du, W., Huang, W., Gu, Z., & Feng, Z. (2021). Microplastics in different tissues of wild crabs at three important fishing grounds in China. *Chemosphere*, *271*(129479), 129479. doi:10.1016/j.chemosphere.2020.129479
88. Baldwin, A. K., Spanjer, A. R., Rosen, M. R., & Thom, T. (2020). Microplastics in Lake Mead National Recreation Area, USA: Occurrence and biological uptake. *PloS One*, *15*(5), e0228896. doi:10.1371/journal.pone.0228896
89. Mercy, F. T., & Alam, A. K. M. R. (2024). Assessment of microplastic contamination in shrimps from the Bay of Bengal and associated human health risk. *Marine Pollution Bulletin*, *201*(116185), 116185. doi:10.1016/j.marpolbul.2024.116185
90. Dambrosio, A., Cometa, S., Capuozzo, F., Ceci, E., Derosa, M., & Quaglia, N. C. (2023). Occurrence and characterization of microplastics in commercial mussels (Mytilus galloprovincialis) from Apulia region (Italy). *Foods (Basel, Switzerland)*, *12*(7). doi:10.3390/foods12071495
91. Reunura, T., & Prommi, T. O. (2022). Detection of microplastics in Litopenaeus vannamei (Penaeidae) and Macrobrachium rosenbergii (Palaemonidae) in cultured pond. *PeerJ*, *10*(e12916), e12916. doi:10.7717/peerj.12916
92. Li, R., Zhang, S., Zhang, L., Yu, K., Wang, S., & Wang, Y. (2020). Field study of the microplastic pollution in sea snails (Ellobium chinense) from mangrove forest and their relationships with microplastics in water/sediment located on the north of Beibu Gulf. *Environmental Pollution (Barking, Essex: 1987)*, *263*(Pt B), 114368. doi:10.1016/j.envpol.2020.114368
93. Addo, S., Boateng, C. M., Diyie, R. L., Duodu, C. P., Ferni, A. K., Williams, E. A., … Nyarko, E. (2022). Occurrence of microplastics in wild oysters (Crassostrea tulipa) from the Gulf of Guinea and their potential human exposure. *Heliyon*, *8*(12), e12255. doi:10.1016/j.heliyon.2022.e12255
94. Patterson, J., Jeyasanta, K. I., Sathish, N., Booth, A. M., & Edward, J. K. P. (2019). Profiling microplastics in the Indian edible oyster, Magallana bilineata collected from the Tuticorin coast, Gulf of Mannar, Southeastern India. *The Science of the Total Environment*, *691*, 727–735. doi:10.1016/j.scitotenv.2019.07.063
95. Wu, F., Wang, Y., Leung, J. Y. S., Huang, W., Zeng, J., Tang, Y., … Cao, L. (2020). Accumulation of microplastics in typical commercial aquatic species: A case study at a productive aquaculture site in China. *The Science of the Total Environment*, *708*(135432), 135432. doi:10.1016/j.scitotenv.2019.135432
96. Corami, F., Rosso, B., Roman, M., Picone, M., Gambaro, A., & Barbante, C. (2020). Evidence of small microplastics (<100 μm) ingestion by Pacific oysters (Crassostrea gigas): A novel method of extraction, purification, and analysis using Micro-FTIR. *Marine Pollution Bulletin*, *160*(111606), 111606. doi:10.1016/j.marpolbul.2020.111606

97. Neves, R. A. F., Guimarães, T. B., & Santos, L. N. (2023). First record of microplastic contamination in the non-native dark false mussel Mytilopsis leucophaeata (Bivalvia: Dreissenidae) in a coastal urban lagoon. *International Journal of Environmental Research and Public Health*, *21*(1). doi:10.3390/ijerph21010044
98. Abd Rahim, N. H., Satyanarayana, B., Ibrahim, Y. S., Not, C., Idris, I., Mohd Jani, J., … Dahdouh-Guebas, F. (2023). Dataset of microplastics in the mangrove brachyuran crabs at Setiu Wetlands, Peninsular Malaysia. *Data in Brief*, *49*(109420), 109420. doi:10.1016/j.dib.2023.109420
99. Aliko, V., Beqiraj, E. G., Qirjo, M., Cani, M., Rama, A., Bego, K., … Faggio, C. (2022). Plastic invasion tolling: First evaluation of microplastics in water and two crab species from the nature reserve lagoonary complex of Kune-Vain, Albania. *The Science of the Total Environment*, *849*(157799), 157799. doi:10.1016/j.scitotenv.2022.157799
100. Yan, M., Li, W., Chen, X., He, Y., Zhang, X., & Gong, H. (2021). A preliminary study of the association between colonization of microorganism on microplastics and intestinal microbiota in shrimp under natural conditions. *Journal of Hazardous Materials*, *408*(124882), 124882. doi:10.1016/j.jhazmat.2020.124882
101. Kazour, M., Jemaa, S., Issa, C., Khalaf, G., & Amara, R. (2019). Microplastics pollution along the Lebanese coast (Eastern Mediterranean Basin): Occurrence in surface water, sediments and biota samples. *The Science of the Total Environment*, *696*(133933), 133933. doi:10.1016/j.scitotenv.2019.133933
102. Narmatha Sathish, M., Immaculate Jeyasanta, K., & Patterson, J. (2020). Monitoring of microplastics in the clam Donax cuneatus and its habitat in Tuticorin coast of Gulf of Mannar (GoM), India. *Environmental Pollution (Barking, Essex: 1987)*, *266*(Pt 1), 115219. doi:10.1016/j.envpol.2020.115219
103. Costa, M. B. da, Otegui, M. B. P., Zamprogno, G. C., Caniçali, F. B., Dos Reis Cozer, C., Pelletier, E., & Graceli, J. B. (2023). Abundance, composition, and distribution of microplastics in intertidal sediment and soft tissues of four species of Bivalvia from Southeast Brazilian urban beaches. *The Science of the Total Environment*, *857*(Pt 3), 159352. doi:10.1016/j.scitotenv.2022.159352
104. Gurjar, U. R., Xavier, M., Nayak, B. B., Ramteke, K., Deshmukhe, G., Jaiswar, A. K., & Shukla, S. P. (2021). Microplastics in shrimps: a study from the trawling grounds of north eastern part of Arabian Sea. *Environmental Science and Pollution Research International*, *28*(35), 48494–48504. doi:10.1007/s11356-021-14121-z
105. Liu, Y., Li, R., Yu, J., Ni, F., Sheng, Y., Scircle, A., … Zhou, Y. (2021). Separation and identification of microplastics in marine organisms by TGA-FTIR-GC/MS: A case study of mussels from coastal China. *Environmental Pollution (Barking, Essex: 1987)*, *272*(115946), 115946. doi:10.1016/j.envpol.2020.115946
106. Keisling, C., Harris, R. D., Blaze, J., Coffin, J., & Byers, J. E. (2020). Low concentrations and low spatial variability of marine microplastics in oysters (Crassostrea virginica) in a rural Georgia estuary. *Marine Pollution Bulletin*, *150*(110672), 110672. doi:10.1016/j.marpolbul.2019.110672
107. Jittalerk, R., & Babel, S. (2023). Microplastic contamination in Thai vinegar crabs (Episesarma mederi), giant mudskippers (Periophthalmodon schlosseri), and their surrounding environment from the Bang Pu mangrove forests, Samut Prakan province, Thailand. *Marine Pollution Bulletin*, *198*, 115849. doi:10.1016/j.marpolbul.2023.115849
108. Renzi, M., Blašković, A., Bernardi, G., & Russo, G. F. (2018). Plastic litter transfer from sediments towards marine trophic webs: A case study on holothurians. *Marine Pollution Bulletin*, *135*, 376–385. doi:10.1016/j.marpolbul.2018.07.038
109. Weir, E. M., Kidd, K. A., Hamilton, B. M., Wu, J., Servos, M. R., Bartlett, A. J., … Gillis, P. L. (2024). Microparticles in wild and caged Biota, sediments, and water relative to large municipal wastewater treatment plant discharges. *Environmental Toxicology and Chemistry*, *43*(5), 1047–1061. doi:10.1002/etc.5836
110. Martinelli, M., Gomiero, A., Guicciardi, S., Frapiccini, E., Strafella, P., Angelini, S., … Colella, S. (2021). Preliminary results on the occurrence and anatomical distribution of microplastics in wild populations of Nephrops norvegicus from the Adriatic Sea. *Environmental Pollution (Barking, Essex: 1987)*, *278*(116872), 116872. doi:10.1016/j.envpol.2021.116872
111. Padmachandran, A. V., Sreethu, N., Nasrin, F., Muthuchamy, M., & Muthukumar, A. (2023). Presence of microplastics in estuarine environment: a case study from Kavvayi and Kumbla backwaters of Malabar Coast, Kerala, India. *Environmental Science and Pollution Research International*. doi:10.1007/s11356-023-26936-z
112. Daniel, D. B., Ashraf, P. M., & Thomas, S. N. (2020). Abundance, characteristics and seasonal variation of microplastics in Indian white shrimps (Fenneropenaeus indicus) from coastal waters

off Cochin, Kerala, India. *The Science of the Total Environment*, *737*(139839), 139839. doi:10.1016/j.scitotenv.2020.139839

113. Bråte, I. L. N., Hurley, R., Iversen, K., Beyer, J., Thomas, K. V., Steindal, C. C., … Lusher, A. (2018). Mytilus spp. as sentinels for monitoring microplastic pollution in Norwegian coastal waters: A qualitative and quantitative study. *Environmental Pollution (Barking, Essex: 1987)*, *243*(Pt A), 383–393. doi:10.1016/j.envpol.2018.08.077
114. Horn, D., Miller, M., Anderson, S., & Steele, C. (2019). Microplastics are ubiquitous on California beaches and enter the coastal food web through consumption by Pacific mole crabs. *Marine Pollution Bulletin*, *139*, 231–237. doi:10.1016/j.marpolbul.2018.12.039
115. Walters, L. J., Busch, S. J., Vermeulen, S., & Craig, C. A. (2024). Entanglement and ingestion of microfibers by the oyster pea crab Zaops ostreum, an endosymbiont of the eastern oyster Crassostrea virginica. *Marine Pollution Bulletin*, *201*(116251), 116251. doi:10.1016/j.marpolbul.2024.116251
116. McGoran, A. R., Clark, P. F., Smith, B. D., & Morritt, D. (2020). High prevalence of plastic ingestion by Eriocheir sinensis and Carcinus maenas (Crustacea: Decapoda: Brachyura) in the Thames Estuary. *Environmental Pollution (Barking, Essex: 1987)*, *265*(Pt A), 114972. doi:10.1016/j.envpol.2020.114972
117. Fernández Severini, M. D., Buzzi, N. S., Forero López, A. D., Colombo, C. V., Chatelain Sartor, G. L., Rimondino, G. N., & Truchet, D. M. (2020). Chemical composition and abundance of microplastics in the muscle of commercial shrimp Pleoticus muelleri at an impacted coastal environment (Southwestern Atlantic). *Marine Pollution Bulletin*, *161*(Pt A), 111700. doi:10.1016/j.marpolbul.2020.111700
118. Valsan, G., Tamrakar, A., & Warrier, A. K. (2024). Microplastics in Scylla Serrata: A baseline study from southwest India. *Marine Pollution Bulletin*, *200*(116109), 116109. doi:10.1016/j.marpolbul.2024.116109
119. Renzi, M., & Blašković, A. (2020). Chemical fingerprint of plastic litter in sediments and holothurians from Croatia: Assessment & relation to different environmental factors. *Marine Pollution Bulletin*, *153*(110994), 110994. doi:10.1016/j.marpolbul.2020.110994
120. Malloggi, C., Nalbone, L., Bartalena, S., Guidi, M., Corradini, C., Foti, A., … Armani, A. (2024). The occurrence of microplastics in Donax trunculus (Mollusca: Bivalvia) collected along the Tuscany coast (Mediterranean sea). *Animals: An Open Access Journal from MDPI*, *14*(4), 618. doi:10.3390/ani14040618
121. Irnidayanti, Y., Soegianto, A., Brabo, A. H., Abdilla, F. M., Indriyasari, K. N., Rahmatin, N. M., … Payus, C. M. (2023). Microplastics in green mussels (Perna viridis) from Jakarta Bay, Indonesia, and the associated hazards to human health posed by their consumption. *Environmental Monitoring and Assessment*, *195*(7), 884. doi:10.1007/s10661-023-11535-9
122. Leung, M. M.-L., Ho, Y.-W., Maboloc, E. A., Lee, C.-H., Wang, Y., Hu, M., … Fang, J. K.-H. (2021). Determination of microplastics in the edible green-lipped mussel Perna viridis using an automated mapping technique of Raman microspectroscopy. *Journal of Hazardous Materials*, *420*(126541), 126541. doi:10.1016/j.jhazmat.2021.126541
123. Rahmatin, N. M., Soegianto, A., Irawan, B., Payus, C. M., Indriyasari, K. N., Marchellina, A., … Irnidayanti, Y. (2023). The spatial distribution and physico-chemical characteristic of microplastics in the sediment and cockle (Anadara granosa) from the coastal waters of East Java, Indonesia, and the health hazards associated with cockle consumption. *Marine Pollution Bulletin*, *198*, 115906. doi:10.1016/j.marpolbul.2023.115906
124. Saldaña-Serrano, M., Bastolla, C. L. V., Mattos, J. J., Lima, D., Freire, T. B., Nogueira, D. J., … Bainy, A. C. D. (2022). Microplastics and linear alkylbenzene levels in oysters Crassostrea gigas driven by sewage contamination at an important aquaculture area of Brazil. *Chemosphere*, *307*(Pt 4), 136039. doi:10.1016/j.chemosphere.2022.136039
125. de Guzman, M. K., Andjelković, M., Jovanović, V., Jung, J., Kim, J., Dailey, L. A., … Ćirković Veličković, T. (2022). Comparative profiling and exposure assessment of microplastics in differently sized Manila clams from South Korea by μFTIR and Nile Red staining. *Marine Pollution Bulletin*, *181*(113846), 113846. doi:10.1016/j.marpolbul.2022.113846
126. Expósito, N., Rovira, J., Sierra, J., Gimenez, G., Domingo, J. L., & Schuhmacher, M. (2022). Levels of microplastics and their characteristics in molluscs from North-West Mediterranean Sea: Human intake. *Marine Pollution Bulletin*, *181*(113843), 113843. doi:10.1016/j.marpolbul.2022.113843
127. Klein, J. R., Beaman, J., Kirkbride, K. P., Patten, C., & Burke da Silva, K. (2022). Microplastics in intertidal water of South Australia and the mussel Mytilus spp.; the contrasting effect of

population on concentration. *The Science of the Total Environment*, *831*(154875), 154875. doi:10.1016/j.scitotenv.2022.154875

128. Mohsen, M., Lin, C., Abdalla, M., Liu, S., & Yang, H. (2023). Microplastics in canned, salt-dried, and instant sea cucumbers sold for human consumption. *Marine Pollution Bulletin*, *192*(115040), 115040. doi:10.1016/j.marpolbul.2023.115040
129. Waddell, E. N., Lascelles, N., & Conkle, J. L. (2020). Microplastic contamination in Corpus Christi Bay blue crabs,*Callinectes sapidus*. *Limnology and Oceanography Letters*, *5*(1), 92–102. doi:10.1002/lol2.10142
130. Not, C., Lui, C. Y. I., & Cannicci, S. (2020). Feeding behavior is the main driver for microparticle intake in mangrove crabs. *Limnology and Oceanography Letters*, *5*(1), 84–91. doi:10.1002/lol2.10143
131. Cozzolino, L., Nicastro, K. R., Lefebvre, S., Corona, L., Froneman, P. W., McQuaid, C., & Zardi, G. I. (2023). The effect of interspecific and intraspecific diversity on microplastic ingestion in two co-occurring mussel species in South Africa. *Marine Pollution Bulletin*, *196*(115649), 115649. doi:10.1016/j.marpolbul.2023.115649
132. Liu, J., Zhu, X., Teng, J., Zhao, J., Li, C., Shan, E., … Wang, Q. (2021). Pollution characteristics of microplastics in mollusks from the coastal area of Yantai, China. *Bulletin of Environmental Contamination and Toxicology*, *107*(4), 693–699. doi:10.1007/s00128-021-03276-7
133. Covernton, G. A., Collicutt, B., Gurney-Smith, H. J., Pearce, C. M., Dower, J. F., Ross, P. S., & Dudas, S. E. (2019). Microplastics in bivalves and their habitat in relation to shellfish aquaculture proximity in coastal British Columbia, Canada. *Aquaculture Environment Interactions*, *11*, 357–374. doi:10.3354/aei00316
134. Tran-Nguyen, Q. A., Nguyen, T. Q., Phan, T. L. T., Van Vo, M., & Trinh-Dang, M. (2023). Abundance of Microplastics in Two Venus Clams (Meretrix lyrata and Paratapes undulatus) from Estuaries in Central Vietnam. *Water*, *15*(7), 1312. doi:10.3390/w15071312
135. Wardlaw, C., & Prosser, R. S. (2020). Investigation of microplastics in freshwater mussels (Lasmigona costata) from the Grand River watershed in Ontario, Canada. *Water, Air, and Soil Pollution*, *231*(8). doi:10.1007/s11270-020-04741-5
136. Ding, J., Li, J., Sun, C., Jiang, F., Ju, P., Qu, L., … He, C. (2019). Detection of microplastics in local marine organisms using a multi-technology system. *Analytical Methods: Advancing Methods and Applications*, *11*(1), 78–87. doi:10.1039/c8ay01974f
137. Hue, H. T. T., Dong, L. K., Hien, T. T., Nguyen, T. N., & Pradit, S. (2021). Assessment of microplastics contamination in commericial clams in the coastal zone of Vietnam. *Applied Ecology and Environmental Research*, *19*(6), 4977–4991. doi:10.15666/aeer/1906_49774991
138. Olivieri, Z., Cesarini, G., Orsini, M., De Santis, S., & Scalici, M. (2022). Uptake of microplastics in the wedge clam Donax trunculus: First evidence from the Mediterranean Sea. *Water*, *14*(24), 4095. doi:10.3390/w14244095
139. Ruangpanupan, N., Ussawarujikulchai, A., Prapagdee, B., & Chavanich, S. (2023). Seasonal variation in the abundance of microplastics in three commercial bivalves from Bandon Bay, Gulf of Thailand. *Marine Pollution Bulletin*, *197*(115600), 115600. doi:10.1016/j.marpolbul.2023.115600

**Fish**

1. Sultana, N., Tista, R. R., Islam, M. S., Begum, M., Islam, S., & Naser, M. N. (2023). Microplastics in freshwater wild and farmed fish species of Bangladesh. *Environmental Science and Pollution Research International*, *30*(28), 72009–72025. doi:10.1007/s11356-023-26512-5
2. Thiele, C. J., Hudson, M. D., Russell, A. E., Saluveer, M., & Sidaoui-Haddad, G. (2021). Microplastics in fish and fishmeal: an emerging environmental challenge? *Scientific Reports*, *11*(1), 2045. doi:10.1038/s41598-021-81499-8
3. Jabeen, K., Su, L., Li, J., Yang, D., Tong, C., Mu, J., & Shi, H. (2017). Microplastics and mesoplastics in fish from coastal and fresh waters of China. *Environmental Pollution (Barking, Essex: 1987)*, *221*, 141–149. doi:10.1016/j.envpol.2016.11.055
4. Sun, X., Li, Q., Shi, Y., Zhao, Y., Zheng, S., Liang, J., … Tian, Z. (2019). Characteristics and retention of microplastics in the digestive tracts of fish from the Yellow Sea. *Environmental Pollution (Barking, Essex: 1987)*, *249*, 878–885. doi:10.1016/j.envpol.2019.01.110
5. Khan, M. B., Urmy, S. Y., Setu, S., Kanta, A. H., Gautam, S., Eti, S. A., … Baten, M. A. (2023). Abundance, distribution and composition of microplastics in sediment and fish species from an Urban River of Bangladesh. *The Science of the Total Environment*, *885*(163876), 163876. doi:10.1016/j.scitotenv.2023.163876
6. Abbasi, A., Sadeghi, P., & Taghizadeh Rahmat Abadi, Z. (2023). Characterization of microplastics in digestive tract of commercial fish species from the Oman Sea. *Marine Pollution Bulletin*, *197*(115769), 115769. doi:10.1016/j.marpolbul.2023.115769

7. Wagner, J., Wang, Z.-M., Ghosal, S., Murphy, M., Wall, S., Cook, A.-M., … Allen, H. (2019). Nondestructive extraction and identification of microplastics from freshwater sport fish stomachs. *Environmental Science & Technology*, *53*(24), 14496–14506. doi:10.1021/acs.est.9b05072
8. Sánchez-Guerrero-Hernández, M. J., González-Fernández, D., Sendra, M., Ramos, F., Yeste, M. P., & González-Ortegón, E. (2023). Contamination from microplastics and other anthropogenic particles in the digestive tracts of the commercial species Engraulis encrasicolus and Sardina pilchardus. *The Science of the Total Environment*, *860*(160451), 160451. doi:10.1016/j.scitotenv.2022.160451
9. Koongolla, J. B., Lin, L., Pan, Y.-F., Yang, C.-P., Sun, D.-R., Liu, S., … Li, H.-X. (2020). Occurrence of microplastics in gastrointestinal tracts and gills of fish from Beibu Gulf, South China Sea. *Environmental Pollution (Barking, Essex: 1987)*, *258*(113734), 113734. doi:10.1016/j.envpol.2019.113734
10. Liu, S., Chen, H., Wang, J., Su, L., Wang, X., Zhu, J., & Lan, W. (2021). The distribution of microplastics in water, sediment, and fish of the Dafeng River, a remote river in China. *Ecotoxicology and Environmental Safety*, *228*(113009), 113009. doi:10.1016/j.ecoenv.2021.113009
11. Carrillo-Barragan, P., Fitzsimmons, C., Lloyd-Hartley, H., Tinlin-Mackenzie, A., Scott, C., & Sugden, H. (2023). Fifty-year study of microplastics ingested by brachyuran and fish larvae in the central English North Sea. *Environmental Pollution (Barking, Essex: 1987)*, 123060. doi:10.1016/j.envpol.2023.123060
12. Lefebvre, C., Saraux, C., Heitz, O., Nowaczyk, A., & Bonnet, D. (2019). Microplastics FTIR characterisation and distribution in the water column and digestive tracts of small pelagic fish in the Gulf of Lions. *Marine Pollution Bulletin*, *142*, 510–519. doi:10.1016/j.marpolbul.2019.03.025
13. Feng, Z., Zhang, T., Li, Y., He, X., Wang, R., Xu, J., & Gao, G. (2019). The accumulation of microplastics in fish from an important fish farm and mariculture area, Haizhou Bay, China. *The Science of the Total Environment*, *696*(133948), 133948. doi:10.1016/j.scitotenv.2019.133948
14. Bahrehmand, M. R., Tabatabaie, T., Hashemi, S. E., Amiri, F., & Pazira, A. R. (2023). Occurrence and spatial distribution of microplastics in sediment and fish along the Persian Gulf-a case study: Bushehr Province, Iran. *Environmental Geochemistry and Health*, *45*(7), 4425–4437. doi:10.1007/s10653-022-01427-1
15. Naidoo, T., Sershen, Thompson, R. C., & Rajkaran, A. (2020). Quantification and characterisation of microplastics ingested by selected juvenile fish species associated with mangroves in KwaZulu-Natal, South Africa. *Environmental Pollution (Barking, Essex: 1987)*, *257*(113635), 113635. doi:10.1016/j.envpol.2019.113635
16. Squillante, J., Scivicco, M., Ariano, A., Nolasco, A., Esposito, F., Cacciola, N. A., … Cirillo, T. (2023). Occurrence of phthalate esters and preliminary data on microplastics in fish from the Tyrrhenian sea (Italy) and impact on human health. *Environmental Pollution (Barking, Essex: 1987)*, *316*(Pt 1), 120664. doi:10.1016/j.envpol.2022.120664
17. Tanaka, K., & Takada, H. (2016). Microplastic fragments and microbeads in digestive tracts of planktivorous fish from urban coastal waters. *Scientific Reports*, *6*(1), 34351. doi:10.1038/srep34351
18. Walkinshaw, C., Tolhurst, T. J., Lindeque, P. K., Thompson, R., & Cole, M. (2022). Detection and characterisation of microplastics and microfibres in fishmeal and soybean meal. *Marine Pollution Bulletin*, *185*(Pt A), 114189. doi:10.1016/j.marpolbul.2022.114189
19. Lusher, A. L., McHugh, M., & Thompson, R. C. (2013). Occurrence of microplastics in the gastrointestinal tract of pelagic and demersal fish from the English Channel. *Marine Pollution Bulletin*, *67*(1–2), 94–99. doi:10.1016/j.marpolbul.2012.11.028
20. Badola, N., Sobhan, F., & Chauhan, J. S. (2023). Microplastics in the River Ganga and its fishes: Study of a Himalayan River. *The Science of the Total Environment*, *901*(165924), 165924. doi:10.1016/j.scitotenv.2023.165924
21. Hossain, M. B., Pingki, F. H., Azad, M. A. S., Nur, A.-A. U., Banik, P., Paray, B. A., … Yu, J. (2023). Microplastics in different tissues of a commonly consumed fish, Scomberomorus guttatus, from a large subtropical estuary: Accumulation, characterization, and contamination assessment. *Biology*, *12*(11), 1422. doi:10.3390/biology12111422
22. Białowąs, M., Jonko-Sobuś, K., Pawlak, J., Polak-Juszczak, L., Dąbrowska, A., & Urban-Malinga, B. (2022). Plastic in digestive tracts and gills of cod and herring from the Baltic Sea. *The Science of the Total Environment*, *822*(153333), 153333. doi:10.1016/j.scitotenv.2022.153333
23. Jaafar, N., Azfaralariff, A., Musa, S. M., Mohamed, M., Yusoff, A. H., & Lazim, A. M. (2021). Occurrence, distribution and characteristics of microplastics in gastrointestinal tract and gills of commercial marine fish from Malaysia. *The Science of the Total Environment*, *799*(149457), 149457. doi:10.1016/j.scitotenv.2021.149457

24. Zhang, F., Xu, J., Zhu, L., Peng, G., Jabeen, K., Wang, X., & Li, D. (2021). Seasonal distributions of microplastics and estimation of the microplastic load ingested by wild caught fish in the East China Sea. *Journal of Hazardous Materials*, *419*(126456), 126456. doi:10.1016/j.jhazmat.2021.126456
25. Ziino, G., Nalbone, L., Giarratana, F., Romano, B., Cincotta, F., & Panebianco, A. (2021). Microplastics in vacuum packages of frozen and glazed icefish (Neosalanx spp.): A freshwater fish intended for human consumption. *Italian Journal of Food Safety*, *10*(4), 9974. doi:10.4081/ijfs.2021.9974
26. Zhao, Y., Sun, X., Li, Q., Shi, Y., Zheng, S., Liang, J., … Tian, Z. (2019). Data on microplastics in the digestive tracts of 19 fish species from the Yellow Sea, China. *Data in Brief*, *25*(103989), 103989. doi:10.1016/j.dib.2019.103989
27. Avio, C. G., Gorbi, S., & Regoli, F. (2015). Experimental development of a new protocol for extraction and characterization of microplastics in fish tissues: First observations in commercial species from Adriatic Sea. *Marine Environmental Research*, *111*, 18–26. doi:10.1016/j.marenvres.2015.06.014
28. Pappoe, C., Palm, L. M. N.-D., Denutsui, D., Boateng, C. M., Danso-Abbeam, H., & Serfor-Armah, Y. (2022). Occurrence of microplastics in gastrointestinal tract of fish from the Gulf of Guinea, Ghana. *Marine Pollution Bulletin*, *182*(113955), 113955. doi:10.1016/j.marpolbul.2022.113955
29. Filgueiras, A. V., Preciado, I., Cartón, A., & Gago, J. (2020). Microplastic ingestion by pelagic and benthic fish and diet composition: A case study in the NW Iberian shelf. *Marine Pollution Bulletin*, *160*(111623), 111623. doi:10.1016/j.marpolbul.2020.111623
30. Rasta, M., Khodadoust, A., Rahimibashar, M. R., Taleshi, M. S., & Sattari, M. (2023). Microplastic pollution in the gastrointestinal tract and gills of some teleost and sturgeon fish from the Caspian Sea, northern Iran. *Environmental Toxicology and Chemistry*, *42*(11), 2453–2465. doi:10.1002/etc.5725
31. Kazour, M., Jemaa, S., El Rakwe, M., Duflos, G., Hermabassiere, L., Dehaut, A., … Amara, R. (2020). Juvenile fish caging as a tool for assessing microplastics contamination in estuarine fish nursery grounds. *Environmental Science and Pollution Research International*, *27*(4), 3548–3559. doi:10.1007/s11356-018-3345-8
32. Klangnurak, W., & Chunniyom, S. (2020). Screening for microplastics in marine fish of Thailand: the accumulation of microplastics in the gastrointestinal tract of different foraging preferences. *Environmental Science and Pollution Research International*, *27*(21), 27161–27168. doi:10.1007/s11356-020-09147-8
33. Atamanalp, M., Köktürk, M., Uçar, A., Duyar, H. A., Özdemir, S., Parlak, V., … Alak, G. (2021). Microplastics in Tissues (Brain, Gill, Muscle and Gastrointestinal) of Mullus barbatus and Alosa immaculata. *Archives of Environmental Contamination and Toxicology*, *81*(3), 460–469. doi:10.1007/s00244-021-00885-5
34. Neves, D., Sobral, P., Ferreira, J. L., & Pereira, T. (2015). Ingestion of microplastics by commercial fish off the Portuguese coast. *Marine Pollution Bulletin*, *101*(1), 119–126. doi:10.1016/j.marpolbul.2015.11.008
35. Saemi-Komsari, M., Esmaeili, H. R., Keshavarzi, B., Abbasi, K., Birami, F. A., Nematollahi, M. J., … Busquets, R. (2023). Characterization of ingested MPs and their relation with growth parameters of endemic and invasive fish from a coastal wetland. *The Science of the Total Environment*, *860*(160495), 160495. doi:10.1016/j.scitotenv.2022.160495
36. Oliveira, A. R., Sardinha-Silva, A., Andrews, P. L. R., Green, D., Cooke, G. M., Hall, S., … Sykes, A. V. (2020). Microplastics presence in cultured and wild-caught cuttlefish, Sepia officinalis. *Marine Pollution Bulletin*, *160*(111553), 111553. doi:10.1016/j.marpolbul.2020.111553
37. Brawn, C., Hamilton, B. M., Savoca, M. S., Bardarson, B., Vermaire, J. C., & Provencher, J. (2023). Suspected anthropogenic microparticle ingestion by Icelandic capelin. *Marine Pollution Bulletin*, *196*(115551), 115551. doi:10.1016/j.marpolbul.2023.115551
38. Riaz, S., Nasreen, S., Burhan, Z., Shafique, S., Alvi, S. A., & Khan, M. A. (2023). Microplastics assessment in Arabian Sea fishes: accumulation, characterization, and method development. *Brazilian Journal of Biology*, *84*, e270694. doi:10.1590/1519-6984.270694
39. Kumkar, P., Gosavi, S. M., Verma, C. R., Pise, M., & Kalous, L. (2021). Big eyes can't see microplastics: Feeding selectivity and eco-morphological adaptations in oral cavity affect microplastic uptake in mud-dwelling amphibious mudskipper fish. *The Science of the Total Environment*, *786*(147445), 147445. doi:10.1016/j.scitotenv.2021.147445
40. Piskuła, P., & Astel, A. M. (2023). Microplastics in commercial fishes and by-catch from selected FAO major fishing areas of the southern Baltic Sea. *Animals: An Open Access Journal from MDPI*, *13*(3). doi:10.3390/ani13030458

41. Mistri, M., Sfriso, A. A., Casoni, E., Nicoli, M., Vaccaro, C., & Munari, C. (2022). Microplastic accumulation in commercial fish from the Adriatic Sea. *Marine Pollution Bulletin*, *174*(113279), 113279. doi:10.1016/j.marpolbul.2021.113279
42. da Costa, I. D., Costa, L. L., da Silva Oliveira, A., de Carvalho, C. E. V., & Zalmon, I. R. (2023). Microplastics in fishes in amazon riverine beaches: Influence of feeding mode and distance to urban settlements. *The Science of the Total Environment*, *863*(160934), 160934. doi:10.1016/j.scitotenv.2022.160934
43. Parker, B., Andreou, D., Pabortsava, K., Barrow, M., Green, I. D., & Britton, J. R. (2022). Microplastic loads within riverine fishes and macroinvertebrates are not predictable from ecological or morphological characteristics. *The Science of the Total Environment*, *839*(156321), 156321. doi:10.1016/j.scitotenv.2022.156321
44. Prata, J. C., da Costa, J. P., Duarte, A. C., & Rocha-Santos, T. (2022). Suspected microplastics in Atlantic horse mackerel fish (Trachurus trachurus) captured in Portugal. *Marine Pollution Bulletin*, *174*(113249), 113249. doi:10.1016/j.marpolbul.2021.113249
45. Zhang, C., Wang, S., Pan, Z., Sun, D., Xie, S., Zhou, A., … Zou, J. (2020). Occurrence and distribution of microplastics in commercial fishes from estuarine areas of Guangdong, South China. *Chemosphere*, *260*(127656), 127656. doi:10.1016/j.chemosphere.2020.127656
46. Wootton, N., Reis-Santos, P., Dowsett, N., Turnbull, A., & Gillanders, B. M. (2021). Low abundance of microplastics in commercially caught fish across southern Australia. *Environmental Pollution (Barking, Essex: 1987)*, *290*(118030), 118030. doi:10.1016/j.envpol.2021.118030
47. Kalaiselvan, K., Pandurangan, P., Velu, R., & Robinson, J. (2022). Occurrence of microplastics in gastrointestinal tracts of planktivorous fish from the Thoothukudi region. *Environmental Science and Pollution Research International*, *29*(29), 44723–44731. doi:10.1007/s11356-022-19033-0
48. Ribeiro-Brasil, D. R. G., Torres, N. R., Picanço, A. B., Sousa, D. S., Ribeiro, V. S., Brasil, L. S., & Montag, L. F. de A. (2020). Contamination of stream fish by plastic waste in the Brazilian Amazon. *Environmental Pollution (Barking, Essex: 1987)*, *266*(Pt 1), 115241. doi:10.1016/j.envpol.2020.115241
49. Chagnon, C., Thiel, M., Antunes, J., Ferreira, J. L., Sobral, P., & Ory, N. C. (2018). Plastic ingestion and trophic transfer between Easter Island flying fish (Cheilopogon rapanouiensis) and yellowfin tuna (Thunnus albacares) from Rapa Nui (Easter Island). *Environmental Pollution (Barking, Essex: 1987)*, *243*(Pt A), 127–133. doi:10.1016/j.envpol.2018.08.042
50. Bessa, F., Barría, P., Neto, J. M., Frias, J. P. G. L., Otero, V., Sobral, P., & Marques, J. C. (2018). Occurrence of microplastics in commercial fish from a natural estuarine environment. *Marine Pollution Bulletin*, *128*, 575–584. doi:10.1016/j.marpolbul.2018.01.044
51. Bilal, M., Ul Hassan, H., Siddique, M. A. M., Khan, W., Gabol, K., Ullah, I., … Arai, T. (2022). Microplastics in the surface water and gastrointestinal tract of Salmo trutta from the mahodand lake, Kalam Swat in Pakistan. *Toxics*, *11*(1), 3. doi:10.3390/toxics11010003
52. Trani, A., Mezzapesa, G., Piscitelli, L., Mondelli, D., Nardelli, L., Belmonte, G., … Zuccaro, M. (2023). Microplastics in water surface and in the gastrointestinal tract of target marine organisms in Salento coastal seas (Italy, Southern Puglia). *Environmental Pollution (Barking, Essex: 1987)*, *316*(Pt 1), 120702. doi:10.1016/j.envpol.2022.120702
53. Wu, J., Yin, X., Liu, Y., Chen, X., Xie, C., Liang, Y., … Jiang, Z. (2022). Seasonal variation and ecological risk assessment of microplastics ingested by economic fishes in Lake Chaohu, China. *The Science of the Total Environment*, *833*(155181), 155181. doi:10.1016/j.scitotenv.2022.155181
54. Kuśmierek, N., & Popiołek, M. (2020). Microplastics in freshwater fish from Central European lowland river (Widawa R., SW Poland). *Environmental Science and Pollution Research International*, *27*(10), 11438–11442. doi:10.1007/s11356-020-08031-9
55. Garcés-Ordóñez, O., Saldarriaga-Vélez, J. F., Espinosa-Díaz, L. F., Patiño, A. D., Cusba, J., Canals, M., … Thiel, M. (2022). Microplastic pollution in water, sediments and commercial fish species from Ciénaga Grande de Santa Marta lagoon complex, Colombian Caribbean. *The Science of the Total Environment*, *829*(154643), 154643. doi:10.1016/j.scitotenv.2022.154643
56. Giani, D., Baini, M., Galli, M., Casini, S., & Fossi, M. C. (2019). Microplastics occurrence in edible fish species (Mullus barbatus and Merluccius merluccius) collected in three different geographical sub-areas of the Mediterranean Sea. *Marine Pollution Bulletin*, *140*, 129–137. doi:10.1016/j.marpolbul.2019.01.005
57. Chen, Y., Shen, Z., Li, G., Wang, K., Cai, X., Xiong, X., & Wu, C. (2022). Factors affecting microplastic accumulation by wild fish: A case study in the Nandu River, South China. *The Science of the Total Environment*, *847*(157486), 157486. doi:10.1016/j.scitotenv.2022.157486
58. Karuppasamy, P. K., Ravi, A., Vasudevan, L., Elangovan, M. P., Dyana Mary, P., Vincent, S. G. T., & Palanisami, T. (2020). Baseline survey of micro and mesoplastics in the gastro-intestinal tract of

commercial fish from Southeast coast of the Bay of Bengal. *Marine Pollution Bulletin*, *153*(110974), 110974. doi:10.1016/j.marpolbul.2020.110974
59. Garnier, Y., Jacob, H., Guerra, A. S., Bertucci, F., & Lecchini, D. (2019). Evaluation of microplastic ingestion by tropical fish from Moorea Island, French Polynesia. *Marine Pollution Bulletin*, *140*, 165–170. doi:10.1016/j.marpolbul.2019.01.038
60. Yagi, M., Kobayashi, T., Maruyama, Y., Hoshina, S., Masumi, S., Aizawa, I., … Shimizu, K. (2022). Microplastic pollution of commercial fishes from coastal and offshore waters in southwestern Japan. *Marine Pollution Bulletin*, *174*(113304), 113304. doi:10.1016/j.marpolbul.2021.113304
61. Khan, L., Ghias, S., Zafar, M. I., Alhodaib, A., Fatima, H., Ur-Rehman, T., … Howari, H. (2022). Exploration of microplastic pollution with particular focus on source identification and spatial patterns in riverine water, sediment and fish of the Swat River, Pakistan. *RSC Advances*, *12*(16), 9556–9566. doi:10.1039/d2ra00319h
62. Matluba, M., Ahmed, M. K., Chowdhury, K. M. A., Khan, N., Ashiq, M. A. R., & Islam, M. S. (2023). The pervasiveness of microplastic contamination in the gastrointestinal tract of fish from the western coast of Bangladesh. *Marine Pollution Bulletin*, *193*(115145), 115145. doi:10.1016/j.marpolbul.2023.115145
63. Cohen-Sánchez, A., Solomando, A., Pinya, S., Tejada, S., Valencia, J. M., Box, A., & Sureda, A. (2023). Microplastic presence in the digestive tract of pearly razorfish Xyrichtys novacula causes oxidative stress in liver tissue. *Toxics*, *11*(4). doi:10.3390/toxics11040365
64. Cocci, P., Gabrielli, S., Pastore, G., Minicucci, M., Mosconi, G., & Palermo, F. A. (2022). Microplastics accumulation in gastrointestinal tracts of Mullus barbatus and Merluccius merluccius is associated with increased cytokine production and signaling. *Chemosphere*, *307*(Pt 3), 135813. doi:10.1016/j.chemosphere.2022.135813
65. Lv, W., Zhou, W., Lu, S., Huang, W., Yuan, Q., Tian, M., … He, D. (2019). Microplastic pollution in rice-fish co-culture system: A report of three farmland stations in Shanghai, China. *The Science of the Total Environment*, *652*, 1209–1218. doi:10.1016/j.scitotenv.2018.10.321
66. Ferreira, G. V. B., Justino, A. K. S., Eduardo, L. N., Schmidt, N., Martins, J. R., Ménard, F., … Lucena-Frédou, F. (2023). Influencing factors for microplastic intake in abundant deep-sea lanternfishes (Myctophidae). *The Science of the Total Environment*, *867*(161478), 161478. doi:10.1016/j.scitotenv.2023.161478
67. Hou, L., McMahan, C. D., McNeish, R. E., Munno, K., Rochman, C. M., & Hoellein, T. J. (2021). A fish tale: a century of museum specimens reveal increasing microplastic concentrations in freshwater fish. *Ecological Applications: A Publication of the Ecological Society of America*, *31*(5), e02320. doi:10.1002/eap.2320
68. López-Martínez, S., Perez-Rubín, C., Gavara, R., Handcock, R. N., & Rivas, M. L. (2022). Presence and implications of plastics in wild commercial fishes in the Alboran Sea (Mediterranean Sea). *The Science of the Total Environment*, *850*(158025), 158025. doi:10.1016/j.scitotenv.2022.158025
69. Robin, R. S., Karthik, R., Purvaja, R., Ganguly, D., Anandavelu, I., Mugilarasan, M., & Ramesh, R. (2020). Holistic assessment of microplastics in various coastal environmental matrices, southwest coast of India. *The Science of the Total Environment*, *703*(134947), 134947. doi:10.1016/j.scitotenv.2019.134947
70. Nelms, S. E., Galloway, T. S., Godley, B. J., Jarvis, D. S., & Lindeque, P. K. (2018). Investigating microplastic trophic transfer in marine top predators. *Environmental Pollution (Barking, Essex: 1987)*, *238*, 999–1007. doi:10.1016/j.envpol.2018.02.016
71. Solomando, A., Cohen-Sánchez, A., Box, A., Montero, I., Pinya, S., & Sureda, A. (2022). Microplastic presence in the pelagic fish, Seriola dumerili, from Balearic Islands (Western Mediterranean), and assessment of oxidative stress and detoxification biomarkers in liver. *Environmental Research*, *212*(Pt C), 113369. doi:10.1016/j.envres.2022.113369
72. Saad, D., & Alamin, H. (2024). The first evidence of microplastic presence in the River Nile in Khartoum, Sudan: Using Nile Tilapia fish as a bio-indicator. *Heliyon*, *10*(1), e23393. doi:10.1016/j.heliyon.2023.e23393
73. Collicutt, B., Juanes, F., & Dudas, S. E. (2019). Microplastics in juvenile Chinook salmon and their nearshore environments on the east coast of Vancouver Island. *Environmental Pollution (Barking, Essex: 1987)*, *244*, 135–142. doi:10.1016/j.envpol.2018.09.137
74. Jeyasanta, K. I., Laju, R. L., Patterson, J., Jayanthi, M., Bilgi, D. S., Sathish, N., & Edward, J. K. P. (2023). Microplastic pollution and its implicated risks in the estuarine environment of Tamil Nadu, India. *The Science of the Total Environment*, *861*(160572), 160572. doi:10.1016/j.scitotenv.2022.160572
75. Parker, B. W., Beckingham, B. A., Ingram, B. C., Ballenger, J. C., Weinstein, J. E., & Sancho, G. (2020). Microplastic and tire wear particle occurrence in fishes from an urban estuary: Influence of

feeding characteristics on exposure risk. *Marine Pollution Bulletin*, *160*(111539), 111539. doi:10.1016/j.marpolbul.2020.111539
76. Blettler, M. C. M., Garello, N., Ginon, L., Abrial, E., Espinola, L. A., & Wantzen, K. M. (2019). Massive plastic pollution in a mega-river of a developing country: Sediment deposition and ingestion by fish (Prochilodus lineatus). *Environmental Pollution (Barking, Essex: 1987)*, *255*(Pt 3), 113348. doi:10.1016/j.envpol.2019.113348
77. Pellini, G., Gomiero, A., Fortibuoni, T., Ferrà, C., Grati, F., Tassetti, A. N., … Scarcella, G. (2018). Characterization of microplastic litter in the gastrointestinal tract of Solea solea from the Adriatic Sea. *Environmental Pollution (Barking, Essex: 1987)*, *234*, 943–952. doi:10.1016/j.envpol.2017.12.038
78. Gedik, K., Eryaşar, A. R., Emanet, M., Şahin, C., & Ceylan, Y. (2023). Monthly microplastics change in European anchovy's (Engraulis encrasicolus) gastrointestinal tract in the Black Sea. *Marine Pollution Bulletin*, *194*(Pt B), 115303. doi:10.1016/j.marpolbul.2023.115303
79. Bhatt, V., Badola, N., & Chauhan, J. S. (2023). Microplastic in fishes: the first report from a Himalayan River - Alaknanda. *Environmental Science and Pollution Research International*. doi:10.1007/s11356-023-30889-8
80. Savoca, S., Capillo, G., Mancuso, M., Bottari, T., Crupi, R., Branca, C., … Spanò, N. (2019). Microplastics occurrence in the Tyrrhenian waters and in the gastrointestinal tract of two congener species of seabreams. *Environmental Toxicology and Pharmacology*, *67*, 35–41. doi:10.1016/j.etap.2019.01.011
81. Khan, F. R., Shashoua, Y., Crawford, A., Drury, A., Sheppard, K., Stewart, K., & Sculthorp, T. (2020). 'the plastic Nile': First evidence of microplastic contamination in fish from the Nile River (Cairo, Egypt). *Toxics*, *8*(2), 22. doi:10.3390/toxics8020022
82. Kılıç, E., & Yücel, N. (2022). Microplastic occurrence in the gastrointestinal tract and gill of bioindicator fish species in the northeastern Mediterranean. *Marine Pollution Bulletin*, *177*(113556), 113556. doi:10.1016/j.marpolbul.2022.113556
83. Ryan, M. G., Watkins, L., & Walter, M. T. (2019). Hudson River juvenile Blueback herring avoid ingesting microplastics. *Marine Pollution Bulletin*, *146*, 935–939. doi:10.1016/j.marpolbul.2019.07.004
84. Hossain, M. S., Sobhan, F., Uddin, M. N., Sharifuzzaman, S. M., Chowdhury, S. R., Sarker, S., & Chowdhury, M. S. N. (2019). Microplastics in fishes from the Northern Bay of Bengal. *The Science of the Total Environment*, *690*, 821–830. doi:10.1016/j.scitotenv.2019.07.065
85. Mancia, A., Chenet, T., Bono, G., Geraci, M. L., Vaccaro, C., Munari, C., … Pasti, L. (2020). Adverse effects of plastic ingestion on the Mediterranean small-spotted catshark (Scyliorhinus canicula). *Marine Environmental Research*, *155*(104876), 104876. doi:10.1016/j.marenvres.2020.104876
86. Galafassi, S., Sighicelli, M., Pusceddu, A., Bettinetti, R., Cau, A., Temperini, M. E., … Volta, P. (2021). Microplastic pollution in perch (Perca fluviatilis, Linnaeus 1758) from Italian south-alpine lakes. *Environmental Pollution (Barking, Essex: 1987)*, *288*(117782), 117782. doi:10.1016/j.envpol.2021.117782
87. Gong, Y., Huang, X., Li, Z., Shen, Y., Li, Y., Zhu, J., & Wu, F. (2023). Plastic ingestion and trophic transfer in an endangered top predator, the longfin mako shark (Isurus paucus), from the tropical western Pacific Ocean. *Environmental Science and Pollution Research International*, *30*(49), 107365–107370. doi:10.1007/s11356-023-25532-5
88. Güven, O., Gökdağ, K., Jovanović, B., & Kıdeyş, A. E. (2017). Microplastic litter composition of the Turkish territorial waters of the Mediterranean Sea, and its occurrence in the gastrointestinal tract of fish. *Environmental Pollution (Barking, Essex: 1987)*, *223*, 286–294. doi:10.1016/j.envpol.2017.01.025
89. Beer, S., Garm, A., Huwer, B., Dierking, J., & Nielsen, T. G. (2018). No increase in marine microplastic concentration over the last three decades – A case study from the Baltic Sea. *The Science of the Total Environment*, *621*, 1272–1279. doi:10.1016/j.scitotenv.2017.10.101
90. Khattab, Y., Mohammadein, A., Al Malki, J. S., Hussien, N. A., & Tantawy, E. M. (2022). Preliminary screening of microplastic contamination in different marine fish species of Taif market, Saudi Arabia. *Open Life Sciences*, *17*(1), 333–343. doi:10.1515/biol-2022-0034
91. Ory, N., Chagnon, C., Felix, F., Fernández, C., Ferreira, J. L., Gallardo, C., … Thiel, M. (2018). Low prevalence of microplastic contamination in planktivorous fish species from the southeast Pacific Ocean. *Marine Pollution Bulletin*, *127*, 211–216. doi:10.1016/j.marpolbul.2017.12.016
92. Borges-Ramírez, M. M., Mendoza-Franco, E. F., Escalona-Segura, G., & Osten, J. R. (2020). Plastic density as a key factor in the presence of microplastic in the gastrointestinal tract of commercial fishes from Campeche Bay, Mexico. *Environmental Pollution (Barking, Essex: 1987)*, *267*(115659), 115659. doi:10.1016/j.envpol.2020.115659
93. Rodríguez-Romeu, O., Constenla, M., Carrassón, M., Campoy-Quiles, M., & Soler-Membrives, A. (2020). Are anthropogenic fibres a real problem for red mullets (Mullus barbatus) from the NW

Mediterranean? *The Science of the Total Environment*, *733*(139336), 139336. doi:10.1016/j.scitotenv.2020.139336
94. Onay, H., Minaz, M., Ak, K., Er, A., Emanet, M., Karslı, B., & Bilgin, S. (2023). Decade of microplastic alteration in the southeastern black sea: An example of seahorse gastrointestinal tracts. *Environmental Research*, *218*(115001), 115001. doi:10.1016/j.envres.2022.115001
95. Buwono, N. R., Risjani, Y., & Soegianto, A. (2022). Oxidative stress responses of microplastic-contaminated Gambusia affinis obtained from the Brantas River in East Java, Indonesia. *Chemosphere*, *293*(133543), 133543. doi:10.1016/j.chemosphere.2022.133543
96. Nithin, A., Sundaramanickam, A., Iswarya, P., & Babu, O. G. (2022). Hazard index of microplastics contamination in various fishes collected off Parangipettai, Southeast coast of India. *Chemosphere*, *307*(Pt 4), 136037. doi:10.1016/j.chemosphere.2022.136037
97. Slootmaekers, B., Catarci Carteny, C., Belpaire, C., Saverwyns, S., Fremout, W., Blust, R., & Bervoets, L. (2019). Microplastic contamination in gudgeons (Gobio gobio) from Flemish rivers (Belgium). *Environmental Pollution (Barking, Essex: 1987)*, *244*, 675–684. doi:10.1016/j.envpol.2018.09.136
98. Pegado, T. de S. E. S., Schmid, K., Winemiller, K. O., Chelazzi, D., Cincinelli, A., Dei, L., & Giarrizzo, T. (2018). First evidence of microplastic ingestion by fishes from the Amazon River estuary. *Marine Pollution Bulletin*, *133*, 814–821. doi:10.1016/j.marpolbul.2018.06.035
99. Garcés-Ordóñez, O., Mejía-Esquivia, K. A., Sierra-Labastidas, T., Patiño, A., Blandón, L. M., & Espinosa Díaz, L. F. (2020). Prevalence of microplastic contamination in the digestive tract of fishes from mangrove ecosystem in Cispata, Colombian Caribbean. *Marine Pollution Bulletin*, *154*(111085), 111085. doi:10.1016/j.marpolbul.2020.111085
100. Pereira, J. M., Rodríguez, Y., Blasco-Monleon, S., Porter, A., Lewis, C., & Pham, C. K. (2020). Microplastic in the stomachs of open-ocean and deep-sea fishes of the North-East Atlantic. *Environmental Pollution (Barking, Essex: 1987)*, *265*(Pt A), 115060. doi:10.1016/j.envpol.2020.115060
101. Walls, L. G., Reusch, T., Clemmesen, C., & Ory, N. C. (2022). Effects of changing environmental conditions on plastic ingestion and feeding ecology of a benthopelagic fish (Gadus morhua) in the Southwest Baltic Sea. *Marine Pollution Bulletin*, *182*(114001), 114001. doi:10.1016/j.marpolbul.2022.114001
102. Bayo, J., Rojo, D., Martínez-Baños, P., López-Castellanos, J., & Olmos, S. (2021). Commercial gilthead seabream (Sparus aurata L.) from the Mar Menor coastal lagoon as hotspots of microplastic accumulation in the digestive system. *International Journal of Environmental Research and Public Health*, *18*(13), 6844. doi:10.3390/ijerph18136844
103. Sánchez-Almeida, R., Hernández-Sánchez, C., Villanova-Solano, C., Díaz-Peña, F. J., Clemente, S., González-Sálamo, J., … Hernández-Borges, J. (2022). Microplastics determination in gastrointestinal tracts of European sea bass (Dicentrarchus labrax) and gilt-head sea bream (Sparus aurata) from Tenerife (Canary Islands, Spain). *Polymers*, *14*(10), 1931. doi:10.3390/polym14101931
104. Anandhan, K., Tharini, K., Thangal, S. H., Yogeshwaran, A., & Muralisankar, T. (2022). Occurrence of microplastics in the gastrointestinal tracts of edible fishes from south Indian rivers. *Bulletin of Environmental Contamination and Toxicology*, *109*(6), 1023–1028. doi:10.1007/s00128-022-03595-3
105. Arias, A. H., Ronda, A. C., Oliva, A. L., & Marcovecchio, J. E. (2019). Evidence of microplastic ingestion by fish from the Bahía Blanca estuary in Argentina, south America. *Bulletin of Environmental Contamination and Toxicology*, *102*(6), 750–756. doi:10.1007/s00128-019-02604-2
106. Herrera, A., Ŝtindlová, A., Martínez, I., Rapp, J., Romero-Kutzner, V., Samper, M. D., … Gómez, M. (2019). Microplastic ingestion by Atlantic chub mackerel (Scomber colias) in the Canary Islands coast. *Marine Pollution Bulletin*, *139*, 127–135. doi:10.1016/j.marpolbul.2018.12.022
107. Collard, F., Gasperi, J., Gilbert, B., Eppe, G., Azimi, S., Rocher, V., & Tassin, B. (2018). Anthropogenic particles in the stomach contents and liver of the freshwater fish Squalius cephalus. *The Science of the Total Environment*, *643*, 1257–1264. doi:10.1016/j.scitotenv.2018.06.313
108. Taghizadeh Rahmat Abadi, Z., Abtahi, B., Grossart, H.-P., & Khodabandeh, S. (2021). Microplastic content of Kutum fish, Rutilus frisii kutum in the southern Caspian Sea. *The Science of the Total Environment*, *752*(141542), 141542. doi:10.1016/j.scitotenv.2020.141542
109. Cohen-Sánchez, A., Solomando, A., Pinya, S., Tejada, S., Valencia, J. M., Box, A., & Sureda, A. (2022). First detection of microplastics in Xyrichtys novacula (Linnaeus 1758) digestive tract from Eivissa Island (Western Mediterranean). *Environmental Science and Pollution Research International*, *29*(43), 65077–65087. doi:10.1007/s11356-022-20298-8

110. Rasta, M., Sattari, M., Taleshi, M. S., & Namin, J. I. (2021). Microplastics in different tissues of some commercially important fish species from Anzali Wetland in the Southwest Caspian Sea, Northern Iran. *Marine Pollution Bulletin*, *169*(112479), 112479. doi:10.1016/j.marpolbul.2021.112479
111. Schirinzi, G. F., Pedà, C., Battaglia, P., Laface, F., Galli, M., Baini, M., … Romeo, T. (2020). A new digestion approach for the extraction of microplastics from gastrointestinal tracts (GITs) of the common dolphinfish (Coryphaena hippurus) from the western Mediterranean Sea. *Journal of Hazardous Materials*, *397*(122794), 122794. doi:10.1016/j.jhazmat.2020.122794
112. Zakeri, M., Naji, A., Akbarzadeh, A., & Uddin, S. (2020). Microplastic ingestion in important commercial fish in the southern Caspian Sea. *Marine Pollution Bulletin*, *160*(111598), 111598. doi:10.1016/j.marpolbul.2020.111598
113. Lusher, Amy L., Hernandez-Milian, G., O'Brien, J., Berrow, S., O'Connor, I., & Officer, R. (2015). Microplastic and macroplastic ingestion by a deep diving, oceanic cetacean: the True's beaked whale Mesoplodon mirus. *Environmental Pollution (Barking, Essex: 1987)*, *199*, 185–191. doi:10.1016/j.envpol.2015.01.023
114. Michishita, S., Gibble, C., Tubbs, C., Felton, R., Gjeltema, J., Lang, J., & Finkelstein, M. (2023). Microplastic in northern anchovies (Engraulis mordax) and common murres (Uria aalge) from the Monterey Bay, California USA - Insights into prevalence, composition, and estrogenic activity. *Environmental Pollution (Barking, Essex: 1987)*, *316*(Pt 2), 120548. doi:10.1016/j.envpol.2022.120548
115. McGoran, A. R., Cowie, P. R., Clark, P. F., McEvoy, J. P., & Morritt, D. (2018). Ingestion of plastic by fish: A comparison of Thames Estuary and Firth of Clyde populations. *Marine Pollution Bulletin*, *137*, 12–23. doi:10.1016/j.marpolbul.2018.09.054
116. Esposito, G., Prearo, M., Renzi, M., Anselmi, S., Cesarani, A., Barcelò, D., … Pastorino, P. (2022). Occurrence of microplastics in the gastrointestinal tract of benthic by-catches from an eastern Mediterranean deep-sea environment. *Marine Pollution Bulletin*, *174*(113231), 113231. doi:10.1016/j.marpolbul.2021.113231
117. Zhang, K., Xiong, X., Hu, H., Wu, C., Bi, Y., Wu, Y., … Liu, J. (2017). Occurrence and characteristics of microplastic pollution in xiangxi bay of Three Gorges Reservoir, China. *Environmental Science & Technology*, *51*(7), 3794–3801. doi:10.1021/acs.est.7b00369
118. Collard, F., Gilbert, B., Compère, P., Eppe, G., Das, K., Jauniaux, T., & Parmentier, E. (2017). Microplastics in livers of European anchovies ( Engraulis encrasicolus , L.). *Environmental Pollution (Barking, Essex: 1987)*, *229*, 1000–1005. doi:10.1016/j.envpol.2017.07.089
119. Ahmed, M., Phukan, B., Talukdar, A., Ahmed, I., Sarma, J., Ali, A., … Xavier, M. (2023). Assessment of microplastic contamination in the gastrointestinal tracts of indigenous fishes from north eastern hill regions of Bhogdoi, a tributary of River Brahmaputra, India. *Environmental Science and Pollution Research International*. doi:10.1007/s11356-023-30821-0
120. Sainio, E., Lehtiniemi, M., & Setälä, O. (2021). Microplastic ingestion by small coastal fish in the northern Baltic Sea, Finland. *Marine Pollution Bulletin*, *172*(112814), 112814. doi:10.1016/j.marpolbul.2021.112814
121. Arafat, S. T., Tanoiri, H., Yokota, M., Nakano, H., Arakawa, H., Terahara, T., & Kobayashi, T. (2023). Microplastic pollution in the gastrointestinal tract of giant river catfish Sperata seenghala (Sykes, 1839) from the Meghna River, Bangladesh. *Environmental Science and Pollution Research International*, *30*(38), 89627–89637. doi:10.1007/s11356-023-28750-z
122. Baalkhuyur, F. M., Bin Dohaish, E.-J. A., Elhalwagy, M. E. A., Alikunhi, N. M., AlSuwailem, A. M., Røstad, A., … Duarte, C. M. (2018). Microplastic in the gastrointestinal tract of fishes along the Saudi Arabian Red Sea coast. *Marine Pollution Bulletin*, *131*, 407–415. doi:10.1016/j.marpolbul.2018.04.040
123. Justino, A. K. S., Ferreira, G. V. B., Schmidt, N., Eduardo, L. N., Fauvelle, V., Lenoble, V., … Lucena-Frédou, F. (2022). The role of mesopelagic fishes as microplastics vectors across the deep-sea layers from the Southwestern Tropical Atlantic. *Environmental Pollution (Barking, Essex: 1987)*, *300*(118988), 118988. doi:10.1016/j.envpol.2022.118988
124. Onay, H., Karslı, B., Minaz, M., & Dalgıç, G. (2023). Seasonal monitoring of microplastic pollution in the Southeast Black Sea: An example of red mullet (Mullus barbatus) gastrointestinal tracts. *Marine Pollution Bulletin*, *191*(114886), 114886. doi:10.1016/j.marpolbul.2023.114886
125. Salazar-Pérez, C., Amezcua, F., Rosales-Valencia, A., Green, L., Pollorena-Melendrez, J. E., Sarmiento-Martínez, M. A., … Barletta, M. (2021). First insight into plastics ingestion by fish in the Gulf of California, Mexico. *Marine Pollution Bulletin*, *171*(112705), 112705. doi:10.1016/j.marpolbul.2021.112705

126. Peters, C. A., Hendrickson, E., Minor, E. C., Schreiner, K., Halbur, J., & Bratton, S. P. (2018). Pyr-GC/MS analysis of microplastics extracted from the stomach content of benthivore fish from the Texas Gulf Coast. *Marine Pollution Bulletin*, *137*, 91–95. doi:10.1016/j.marpolbul.2018.09.049
127. Andreas, Hadibarata, T., Sathishkumar, P., Prasetia, H., Hikmat, Pusfitasari, E. D., … Sari, A. A. (2021). Microplastic contamination in the Skipjack Tuna (Euthynnus affinis) collected from Southern Coast of Java, Indonesia. *Chemosphere*, *276*(130185), 130185. doi:10.1016/j.chemosphere.2021.130185
128. Bottari, T., Savoca, S., Mancuso, M., Capillo, G., GiuseppePanarello, G., MartinaBonsignore, M., … Fazio, E. (2019). Plastics occurrence in the gastrointestinal tract of Zeus faber and Lepidopus caudatus from the Tyrrhenian Sea. *Marine Pollution Bulletin*, *146*, 408–416. doi:10.1016/j.marpolbul.2019.07.003
129. Gurjar, U. R., Xavier, K. A. M., Shukla, S. P., Deshmukhe, G., Jaiswar, A. K., & Nayak, B. B. (2021). Incidence of microplastics in gastrointestinal tract of golden anchovy (Coilia dussumieri) from north east coast of Arabian Sea: The ecological perspective. *Marine Pollution Bulletin*, *169*(112518), 112518. doi:10.1016/j.marpolbul.2021.112518
130. Pinho, I., Amezcua, F., Rivera, J. M., Green-Ruiz, C., Piñón-Colin, T. de J., & Wakida, F. (2022). First report of plastic contamination in batoids: Plastic ingestion by Haller's Round Ray (Urobatis halleri) in the Gulf of California. *Environmental Research*, *211*(113077), 113077. doi:10.1016/j.envres.2022.113077
131. Valente, T., Pelamatti, T., Avio, C. G., Camedda, A., Costantini, M. L., de Lucia, G. A., … Matiddi, M. (2022). One is not enough: Monitoring microplastic ingestion by fish needs a multispecies approach. *Marine Pollution Bulletin*, *184*(114133), 114133. doi:10.1016/j.marpolbul.2022.114133
132. Kılıç, E. (2022). Microplastic ingestion evidence by economically important farmed fish species from Turkey. *Marine Pollution Bulletin*, *183*(114097), 114097. doi:10.1016/j.marpolbul.2022.114097
133. Koraltan, İ., Mavruk, S., & Güven, O. (2022). Effect of biological and environmental factors on microplastic ingestion of commercial fish species. *Chemosphere*, *303*(Pt 2), 135101. doi:10.1016/j.chemosphere.2022.135101
134. Rios-Fuster, B., Alomar, C., Paniagua González, G., Garcinuño Martínez, R. M., Soliz Rojas, D. L., Fernández Hernando, P., & Deudero, S. (2022). Assessing microplastic ingestion and occurrence of bisphenols and phthalates in bivalves, fish and holothurians from a Mediterranean marine protected area. *Environmental Research*, *214*(Pt 3), 114034. doi:10.1016/j.envres.2022.114034
135. Garcia, A. G., Suárez, D. C., Li, J., & Rotchell, J. M. (2021). A comparison of microplastic contamination in freshwater fish from natural and farmed sources. *Environmental Science and Pollution Research International*, *28*(12), 14488–14497. doi:10.1007/s11356-020-11605-2

**Drinking Water**

1. Pittroff, M., Müller, Y. K., Witzig, C. S., Scheurer, M., Storck, F. R., & Zumbülte, N. (2021). Microplastic analysis in drinking water based on fractionated filtration sampling and Raman microspectroscopy. *Environmental Science and Pollution Research International*, *28*(42), 59439–59451. doi:10.1007/s11356-021-12467-y
2. Shruti, V. C., Kutralam-Muniasamy, G., Pérez-Guevara, F., Roy, P. D., & Elizalde-Martínez, I. (2022). Free, but not microplastic-free, drinking water from outdoor refill kiosks: A challenge and a wake-up call for urban management. *Environmental Pollution (Barking, Essex: 1987)*, *309*(119800), 119800. doi:10.1016/j.envpol.2022.119800
3. Qian, N., Gao, X., Lang, X., Deng, H., Bratu, T. M., Chen, Q., … Min, W. (2024). Rapid single-particle chemical imaging of nanoplastics by SRS microscopy. *Proceedings of the National Academy of Sciences of the United States of America*, *121*(3), e2300582121. doi:10.1073/pnas.2300582121
4. Yadav, H., Sethulekshmi, S., & Shriwastav, A. (2022). Estimation of microplastic exposure via the composite sampling of drinking water, respirable air, and cooked food from Mumbai, India. *Environmental Research*, *214*(Pt 1), 113735. doi:10.1016/j.envres.2022.113735
5. Oni, B. A., & Sanni, S. E. (2022). Occurrence of microplastics in borehole drinking water and sediments in Lagos, Nigeria. *Environmental Toxicology and Chemistry*, *41*(7), 1721–1731. doi:10.1002/etc.5350
6. Buyukunal, S. K., Koluman, A., & Muratoglu, K. (2023). Microplastic pollution of drinking water in a metropolis. *Journal of Water and Health*, *21*(6), 687–701. doi:10.2166/wh.2023.265
7. Bäuerlein, P. S., Hofman-Caris, R. C. H. M., Pieke, E. N., & Ter Laak, T. L. (2022). Fate of microplastics in the drinking water production. *Water Research*, *221*(118790), 118790. doi:10.1016/j.watres.2022.118790
8. Kirstein, I. V., Hensel, F., Gomiero, A., Iordachescu, L., Vianello, A., Wittgren, H. B., & Vollertsen, J. (2021). Drinking plastics? - Quantification and qualification of microplastics in drinking water

distribution systems by μFTIR and Py-GCMS. *Water Research*, *188*(116519), 116519. doi:10.1016/j.watres.2020.116519
9. Dronjak, L., Exposito, N., Rovira, J., Florencio, K., Emiliano, P., Corzo, B., … Sierra, J. (2022). Screening of microplastics in water and sludge lines of a drinking water treatment plant in Catalonia, Spain. *Water Research*, *225*(119185), 119185. doi:10.1016/j.watres.2022.119185
10. Shi, J., Dong, Y., Shi, Y., Yin, T., He, W., An, T., … Lin, H. (2022). Groundwater antibiotics and microplastics in a drinking-water source area, northern China: Occurrence, spatial distribution, risk assessment, and correlation. *Environmental Research*, *210*(112855), 112855. doi:10.1016/j.envres.2022.112855
11. Chanpiwat, P., & Damrongsiri, S. (2021). Abundance and characteristics of microplastics in freshwater and treated tap water in Bangkok, Thailand. *Environmental Monitoring and Assessment*, *193*(5), 258. doi:10.1007/s10661-021-09012-2
12. Panno, S. V., Kelly, W. R., Scott, J., Zheng, W., McNeish, R. E., Holm, N., … Baranski, E. L. (2019). Microplastic contamination in Karst groundwater systems. *Ground Water*, *57*(2), 189–196. doi:10.1111/gwat.12862
13. Altunışık, A. (2023). Microplastic pollution and human risk assessment in Turkish bottled natural and mineral waters. *Environmental Science and Pollution Research International*, *30*(14), 39815–39825. doi:10.1007/s11356-022-25054-6
14. Praveena, S. M., Shamsul Ariffin, N. I., & Nafisyah, A. L. (2022). Microplastics in Malaysian bottled water brands: Occurrence and potential human exposure. *Environmental Pollution (Barking, Essex: 1987)*, *315*(120494), 120494. doi:10.1016/j.envpol.2022.120494
15. Almaiman, L., Aljomah, A., Bineid, M., Aljeldah, F. M., Aldawsari, F., Liebmann, B., … Alarfaj, R. (2021). The occurrence and dietary intake related to the presence of microplastics in drinking water in Saudi Arabia. *Environmental Monitoring and Assessment*, *193*(7), 390. doi:10.1007/s10661-021-09132-9
16. Dalmau-Soler, J., Ballesteros-Cano, R., Boleda, M. R., Paraira, M., Ferrer, N., & Lacorte, S. (2021). Microplastics from headwaters to tap water: occurrence and removal in a drinking water treatment plant in Barcelona Metropolitan area (Catalonia, NE Spain). *Environmental Science and Pollution Research International*, *28*(42), 59462–59472. doi:10.1007/s11356-021-13220-1
17. Mohan, M., Gaonkar, A. A., Pandyanda Nanjappa, D., K, K., Vittal, R., Chakraborty, A., & Chakraborty, G. (2023). Screening for microplastics in drinking water and its toxicity profiling in zebrafish. *Chemosphere*, *341*(139882), 139882. doi:10.1016/j.chemosphere.2023.139882
18. Zhou, G., Wu, Q., Wei, X.-F., Chen, C., Ma, J., Crittenden, J. C., & Liu, B. (2023). Tracing microplastics in rural drinking water in Chongqing, China: Their presence and pathways from source to tap. *Journal of Hazardous Materials*, *459*, 132206. doi:10.1016/j.jhazmat.2023.132206
19. Pivokonský, M., Pivokonská, L., Novotná, K., Čermáková, L., & Klimtová, M. (2020). Occurrence and fate of microplastics at two different drinking water treatment plants within a river catchment. *The Science of the Total Environment*, *741*(140236), 140236. doi:10.1016/j.scitotenv.2020.140236
20. Negrete Velasco, A., Ramseier Gentile, S., Zimmermann, S., Le Coustumer, P., & Stoll, S. (2023). Contamination and removal efficiency of microplastics and synthetic fibres in a conventional drinking water treatment plant in Geneva, Switzerland. *The Science of the Total Environment*, *880*(163270), 163270. doi:10.1016/j.scitotenv.2023.163270
21. Shen, M., Zeng, Z., Wen, X., Ren, X., Zeng, G., Zhang, Y., & Xiao, R. (2021). Presence of microplastics in drinking water from freshwater sources: the investigation in Changsha, China. *Environmental Science and Pollution Research International*, *28*(31), 42313–42324. doi:10.1007/s11356-021-13769-x
22. Pérez-Guevara, F., Roy, P. D., Elizalde-Martínez, I., Kutralam-Muniasamy, G., & Shruti, V. C. (2022). Human exposure to microplastics from urban decentralized pay-to-fetch drinking-water refill kiosks. *The Science of the Total Environment*, *848*(157722), 157722. doi:10.1016/j.scitotenv.2022.157722
23. Wu, B., Li, L.-W., Zu, Y.-X., Nan, J., Chen, X.-Q., Sun, K., & Li, Z.-L. (2022). Microplastics contamination in groundwater of a drinking-water source area, northern China. *Environmental Research*, *214*(Pt 3), 114048. doi:10.1016/j.envres.2022.114048
24. Adib, D., Mafigholami, R., & Tabeshkia, H. (2021). Identification of microplastics in conventional drinking water treatment plants in Tehran, Iran. *Journal of Environmental Health Science & Engineering*, *19*(2), 1817–1826. doi:10.1007/s40201-021-00737-3

25. Mu, H., Wang, Y., Zhang, H., Guo, F., Li, A., Zhang, S., … Liu, T. (2022). High abundance of microplastics in groundwater in Jiaodong Peninsula, China. *The Science of the Total Environment*, *839*(156318), 156318. doi:10.1016/j.scitotenv.2022.156318
26. Tong, H., Jiang, Q., Hu, X., & Zhong, X. (2020). Occurrence and identification of microplastics in tap water from China. *Chemosphere*, *252*(126493), 126493. doi:10.1016/j.chemosphere.2020.126493
27. Li, H., Zhu, L., Ma, M., Wu, H., An, L., & Yang, Z. (2023). Occurrence of microplastics in commercially sold bottled water. *The Science of the Total Environment*, *867*(161553), 161553. doi:10.1016/j.scitotenv.2023.161553
28. Johnson, A. C., Ball, H., Cross, R., Horton, A. A., Jürgens, M. D., Read, D. S., … Svendsen, C. (2020). Identification and quantification of microplastics in potable water and their sources within water treatment works in England and Wales. *Environmental Science & Technology*, *54*(19), 12326–12334. doi:10.1021/acs.est.0c03211
29. Madejski, G. R., Ahmad, S. D., Musgrave, J., Flax, J., Madejski, J. G., Rowley, D. A., … McGrath, J. L. (2020). Silicon nanomembrane filtration and imaging for the evaluation of microplastic entrainment along a municipal water delivery route. *Sustainability*, *12*(24), 10655. doi:10.3390/su122410655
30. Yang, X., Xu, X., Zhou, Y., Yao, Y., Shen, C., & Liu, J. (2023). Longitudinal and vertical distribution of microplastics in various pipe scales in an operating drinking water distribution system. *Journal of Hazardous Materials*, *459*(132108), 132108. doi:10.1016/j.jhazmat.2023.132108
31. Tse, Y.-T., Chan, S. M.-N., & Sze, E. T.-P. (2022). Quantitative assessment of full size microplastics in bottled and tap water samples in Hong Kong. *International Journal of Environmental Research and Public Health*, *19*(20), 13432. doi:10.3390/ijerph192013432
32. An, X., Li, W., Lan, J., & Adnan, M. (2022). Preliminary study on the distribution, source, and ecological risk of typical microplastics in Karst groundwater in guizhou province, China. *International Journal of Environmental Research and Public Health*, *19*(22), 14751. doi:10.3390/ijerph192214751
33. Shruti, V. C., Pérez-Guevara, F., & Kutralam-Muniasamy, G. (2020). Metro station free drinking water fountain- A potential 'microplastics hotspot' for human consumption. *Environmental Pollution (Barking, Essex: 1987)*, *261*(114227), 114227. doi:10.1016/j.envpol.2020.114227
34. Maurizi, L., Iordachescu, L., Kirstein, I. V., Nielsen, A. H., & Vollertsen, J. (2023). It matters how we measure - Quantification of microplastics in drinking water by μFTIR and μRaman. *Heliyon*, *9*(9), e20119. doi:10.1016/j.heliyon.2023.e20119
35. la Cecilia, D., Philipp, M., Kaegi, R., Schirmer, M., & Moeck, C. (2023). Microplastics attenuation from surface water to drinking water: Impact of treatment and managed aquifer recharge - and identification uncertainties. *The Science of the Total Environment*, *908*(168378), 168378. doi:10.1016/j.scitotenv.2023.168378
36. Kankanige, D., & Babel, S. (2020). Smaller-sized micro-plastics (MPs) contamination in single-use PET-bottled water in Thailand. *The Science of the Total Environment*, *717*(137232), 137232. doi:10.1016/j.scitotenv.2020.137232
37. Taghipour, H., Ghayebzadeh, M., Ganji, F., Mousavi, S., & Azizi, N. (2023). Tracking microplastics contamination in drinking water in Zahedan, Iran: From source to consumption taps. *The Science of the Total Environment*, *872*(162121), 162121. doi:10.1016/j.scitotenv.2023.162121
38. Yang, J., Monnot, M., Sun, Y., Asia, L., Wong-Wah-Chung, P., Doumenq, P., & Moulin, P. (2023). Microplastics in different water samples (seawater, freshwater, and wastewater): Removal efficiency of membrane treatment processes. *Water Research*, *232*(119673), 119673. doi:10.1016/j.watres.2023.119673
39. Gálvez-Blanca, V., Edo, C., González-Pleiter, M., Albentosa, M., Bayo, J., Beiras, R., … Rosal, R. (2023). Occurrence and size distribution study of microplastics in household water from different cities in continental Spain and the Canary Islands. *Water Research*, *238*, 120044. doi:10.1016/j.watres.2023.120044
40. Crosta, A., Parolini, M., & De Felice, B. (2023). Microplastics contamination in nonalcoholic beverages from the Italian market. *International Journal of Environmental Research and Public Health*, *20*(5). doi:10.3390/ijerph20054122
41. Samandra, S., Mescall, O. J., Plaisted, K., Symons, B., Xie, S., Ellis, A. V., & Clarke, B. O. (2022). Assessing exposure of the Australian population to microplastics through bottled water consumption. *The Science of the Total Environment*, *837*(155329), 155329. doi:10.1016/j.scitotenv.2022.155329

42. Islam, M. S., Islam, Z., Jamal, A. H. M. S. I. M., Momtaz, N., & Beauty, S. A. (2023). Removal efficiencies of microplastics of the three largest drinking water treatment plants in Bangladesh. *The Science of the Total Environment*, *895*(165155), 165155. doi:10.1016/j.scitotenv.2023.165155
43. Altunışık, A. (2023). Prevalence of microplastics in commercially sold soft drinks and human risk assessment. *Journal of Environmental Management*, *336*(117720), 117720. doi:10.1016/j.jenvman.2023.117720
44. Mukotaka, A., Kataoka, T., & Nihei, Y. (2021). Rapid analytical method for characterization and quantification of microplastics in tap water using a Fourier-transform infrared microscope. *The Science of the Total Environment*, *790*(148231), 148231. doi:10.1016/j.scitotenv.2021.148231
45. Patil, S., Bafana, A., Krishnamurthi, K., & Sivanesan, S. (2024). Estimated exposure to microplastics through national and local brands of bottled water in Central India. *Environmental Monitoring and Assessment*, *196*(3). doi:10.1007/s10661-024-12387-7
46. Nacaratte, F., Cuevas, P., Becerra-Herrera, M., & Manzano, C. A. (2023). Early screening of suspected microplastics in bottled water in the Santiago Metropolitan Region of Chile. *Environmental Pollution (Barking, Essex: 1987)*, *334*(122118), 122118. doi:10.1016/j.envpol.2023.122118
47. Pivokonsky, M., Cermakova, L., Novotna, K., Peer, P., Cajthaml, T., & Janda, V. (2018). Occurrence of microplastics in raw and treated drinking water. *The Science of the Total Environment*, *643*, 1644–1651. doi:10.1016/j.scitotenv.2018.08.102
48. Mason, S. A., Welch, V. G., & Neratko, J. (2018). Synthetic polymer contamination in bottled water. *Frontiers in Chemistry*, *6*, 407. doi:10.3389/fchem.2018.00407
49. Pavithra, K., Vairaperumal, T., Ks, V., Mukhopadhyay, M., Malar, P., & Chakraborty, P. (2024). Microplastics in packaged water, community stored water, groundwater, and surface water in rivers of Tamil Nadu after the COVID-19 pandemic outbreak. *Journal of Environmental Management*, *356*(120361), 120361. doi:10.1016/j.jenvman.2024.120361
50. Han, Z., Jiang, J., Xia, J., Yan, C., & Cui, C. (2024). Occurrence and fate of microplastics from a water source to two different drinking water treatment plants in a megacity in eastern China. *Environmental Pollution (Barking, Essex: 1987)*, *346*(123546), 123546. doi:10.1016/j.envpol.2024.123546
51. Schymanski, D., Goldbeck, C., Humpf, H.-U., & Fürst, P. (2018). Analysis of microplastics in water by micro-Raman spectroscopy: Release of plastic particles from different packaging into mineral water. *Water Research*, *129*, 154–162. doi:10.1016/j.watres.2017.11.011
52. Oßmann, B. E., Sarau, G., Holtmannspötter, H., Pischetsrieder, M., Christiansen, S. H., & Dicke, W. (2018). Small-sized microplastics and pigmented particles in bottled mineral water. *Water Research*, *141*, 307–316. doi:10.1016/j.watres.2018.05.027
53. Negrete Velasco, A., Ramseier Gentile, S., Zimmermann, S., & Stoll, S. (2022). Contamination and removal efficiency of microplastics and synthetic fibres in a conventional drinking water treatment plant. *Frontiers in Water*, *4*. doi:10.3389/frwa.2022.835451
54. Dalmau-Soler, J., Ballesteros-Cano, R., Ferrer, N., Boleda, M. R., & Lacorte, S. (2022). Microplastics throughout a tap water supply network. *Water and Environment Journal: The Journal*, *36*(2), 292–298. doi:10.1111/wej.12766
55. Feld, L., Silva, V. H. da, Murphy, F., Hartmann, N. B., & Strand, J. (2021). A study of microplastic particles in Danish tap water. *Water*, *13*(15), 2097. doi:10.3390/w13152097
56. Alvim, C. B., Bes-Piá, M. A., Mendoza-Roca, J. A., & Alonso-Molina, J. L. (2023). Identification of microfibers in drinking water with Nile Red. Limitations and strengths. *Journal of Environmental Chemical Engineering*, *11*(3), 109697. doi:10.1016/j.jece.2023.109697
57. Barbier, J.-S., Dris, R., Lecarpentier, C., Raymond, V., Delabre, K., Thibert, S., … Gasperi, J. (2022). Microplastic occurrence after conventional and nanofiltration processes at drinking water treatment plants: Preliminary results. *Frontiers in Water*, *4*. doi:10.3389/frwa.2022.886703
58. Sultan, M., Al-Ahmady, K., & Mhemid, R. K. (2023). Microplastics evaluation in tap water in left side districts of Mosul city, Iraq. *Inżynieria Ekologiczna*, *24*(8), 353–362. doi:10.12911/22998993/166312
59. Radityaningrum, A., Trihadiningrum, Y., & Soedjono, E. (2023). Performance of conventional drinking water treatment plants in removing microplastics in east java, Indonesia. *Inżynieria Ekologiczna*, *24*(6), 129–143. doi:10.12911/22998993/162785
60. Hossain, M. B., Yu, J., Banik, P., Noman, M. A., Nur, A.-A. U., Haque, M. R., … Arai, T. (2023). First evidence of microplastics and their characterization in bottled drinking water from a developing country. *Frontiers in Environmental Science*, *11*. doi:10.3389/fenvs.2023.1232931
61. Sharifi, H., & Movahedian Attar, H. (2022). Identification, quantification, and evaluation of microplastics removal efficiency in a water treatment plant (A case study in Iran). *Air Soil and Water Research*, *15*, 117862212211349. doi:10.1177/11786221221134945

62. Li, Y., Meng, Y., Qin, L., Shen, M., Qin, T., Chen, X., … Duan, X. (2023). Occurrence and removal efficiency of microplastics in four drinking water treatment plants in Zhengzhou, China. *Water*, *16*(1), 131. doi:10.3390/w16010131
63. Ferraz, M., Bauer, A. L., Valiati, V. H., & Schulz, U. H. (2020). Microplastic concentrations in raw and drinking water in the Sinos River, Southern Brazil. *Water*, *12*(11), 3115. doi:10.3390/w12113115
64. Swanepoel, A., Preez, H. du, & Bouwman, H. (2023). A baseline study on the prevalence of microplastics in South African drinking water: from source to distribution. *Water S. A.*, *49*(4 October). doi:10.17159/wsa/2023.v49.i4.3998
65. Radityaningrum, A. D., Trihadiningrum, Y., Mar'atusholihah, Soedjono, E. S., & Herumurti, W. (2021). Microplastic contamination in water supply and the removal efficiencies of the treatment plants: A case of Surabaya City, Indonesia. *Journal of Water Process Engineering*, *43*(102195), 102195. doi:10.1016/j.jwpe.2021.102195
66. Wibuloutai, J., Thongkum, W., Khiewkhern, S., Thunyasirinon, C., & Prathumchai, N. (2023). Microplastics and nanoplastics contamination in raw and treated water. *Water Science & Technology: Water Supply*. doi:10.2166/ws.2023.116
67. Babel, S., & Dork, H. (2021). Identification of micro-plastic contamination in drinking water treatment plants in Phnom Penh, Cambodia. *Journal of Engineering and Technological Sciences*, *53*(3), 210307. doi:10.5614/j.eng.technol.sci.2021.53.3.7
68. Alonso-Vázquez, P., Luján-Facundo, M.-J., Cuartas-Uribe, B., Bes-Piá, A., Alonso-Molina, J.-L., & Mendoza-Roca, J.-A. (2023). Advances in analysis of microplastics in drinking water treatment plants. Fluorescence techniques using iDye Pink. *Environmental Technology & Innovation*, *32*(103430), 103430. doi:10.1016/j.eti.2023.103430
69. Hernandez, L. M., Farner, J. M., Claveau-Mallet, D., Okshevsky, M., Jahandideh, H., Matthews, S., … Tufenkji, N. (2023). Optimizing the concentration of Nile red for screening of microplastics in drinking water. *ACS ES&T Water*, *3*(4), 1029–1038. doi:10.1021/acsestwater.2c00503
70. Kankanige, D., & Babel, S. (2021). Contamination by ≥6.5 μm-sized microplastics and their removability in a conventional water treatment plant (WTP) in Thailand. *Journal of Water Process Engineering*, *40*(101765), 101765. doi:10.1016/j.jwpe.2020.101765
71. Nizamali, J., Mintenig, S. M., & Koelmans, A. A. (2023). Assessing microplastic characteristics in bottled drinking water and air deposition samples using laser direct infrared imaging. *Journal of Hazardous Materials*, *441*(129942), 129942. doi:10.1016/j.jhazmat.2022.129942
72. Aleksander-Kwaterczak, U., Gaj, D., Stelmach, A., & Wróbel, T. P. (2023). Investigating the content of microplastics and other extraneous particles in Polish bottled water. *Geology, Geophysics and Environment*, *49*(4), 335–353. doi:10.7494/geol.2023.49.4.335
73. Kankanige, D., & Babel, S. (2020). Identification of micro-plastics (MPs) in conventional tap water sourced from Thailand. *Journal of Engineering and Technological Sciences*, *52*(1), 95–107. doi:10.5614/j.eng.technol.sci.2020.52.1.7
74. Zhou, X.-J., Wang, J., Li, H.-Y., Zhang, H.-M., Hua-Jiang, & Zhang, D. L. (2021). Microplastic pollution of bottled water in China. *Journal of Water Process Engineering*, *40*(101884), 101884. doi:10.1016/j.jwpe.2020.101884
75. Lam, T. W. L., Ho, H. T., Ma, A. T. H., & Fok, L. (2020). Microplastic contamination of surface water-sourced tap water in Hong Kong—A preliminary study. *Applied Sciences (Basel, Switzerland)*, *10*(10), 3463. doi:10.3390/app10103463
76. Zhang, M., Li, J., Ding, H., Ding, J., Jiang, F., Ding, N. X., & Sun, C. (2020). Distribution characteristics and influencing factors of microplastics in urban tap water and water sources in Qingdao, China. *Analytical Letters*, *53*(8), 1312–1327. doi:10.1080/00032719.2019.1705476
77. Weisser, J., Beer, I., Hufnagl, B., Hofmann, T., Lohninger, H., Ivleva, N. P., & Glas, K. (2021). From the well to the Bottle: Identifying sources of microplastics in mineral water. *Water*, *13*(6), 841. doi:10.3390/w13060841
78. Makhdoumi, P., Amin, A. A., Karimi, H., Pirsaheb, M., Kim, H., & Hossini, H. (2021). Occurrence of microplastic particles in the most popular Iranian bottled mineral water brands and an assessment of human exposure. *Journal of Water Process Engineering*, *39*(101708), 101708. doi:10.1016/j.jwpe.2020.101708
79. Okoffo, E. D., & Thomas, K. V. (2023). Quantitative analysis of nanoplastics in environmental and potable waters by pyrolysis-gas chromatography-mass spectrometry. *Journal of Hazardous Materials*, *464*(133013), 133013. doi:10.1016/j.jhazmat.2023.133013
80. Halfar, J., Brožová, K., Placová, K., & Kyncl, M. (2023, December 1). Determining the presence of micro-particles in drinking water in the Czech Republic—an exploratory study focusing on microplastics and additives. *The 4th International Conference on Advances in Environmental*

*Engineering*. Presented at the The 4th International Conference on Advances in Environmental Engineering. doi:10.3390/engproc2023057016

81. Semmouri, I., Vercauteren, M., Van Acker, E., Pequeur, E., Asselman, J., & Janssen, C. (2022). Presence of microplastics in drinking water from different freshwater sources in Flanders (Belgium), an urbanized region in Europe. *International Journal of Food Contamination*, *9*(1). doi:10.1186/s40550-022-00091-8
82. Zainuddin, Z., & Syuhada. (2020). Study of analysis method on microplastic identification in bottled drinking water. *Macromolecular Symposia*, *391*(1), 1900195. doi:10.1002/masy.201900195
83. Sultan HH, Al-Aadhami MAWS, Baqer NN. Detection of Microplastics in Drinking Water Treatment Plants in Baghdad City/Iraq. *Pollution*. 2023;9(4):1838-1849. doi:10.22059/poll.2023.359382.1913

**Meat**

1. Bai, C.-L., Xu, T.-T., Guo, Y., & Li, H.-T. (2022). A rapid method for extracting microplastics from oily food samples. *Analytical Methods: Advancing Methods and Applications*, *14*(36), 3529–3538. doi:10.1039/d2ay00792d
2. Milne, M. H., De Frond, H., Rochman, C. M., Mallos, N. J., Leonard, G. H., & Baechler, B. R. (2023). Exposure of U.S. adults to microplastics from commonly-consumed proteins. *Environmental Pollution (Barking, Essex: 1987)*, 123233. doi:10.1016/j.envpol.2023.123233
3. Bilal, M., Taj, M., Ul Hassan, H., Yaqub, A., Shah, M. I. A., Sohail, M., … Arai, T. (2023). First report on microplastics quantification in poultry chicken and potential human health risks in Pakistan. *Toxics*, *11*(7). doi:10.3390/toxics11070612
4. Huerta Lwanga, E., Mendoza Vega, J., Ku Quej, V., Chi, J. de L. A., Sanchez del Cid, L., Chi, C., … Geissen, V. (2017). Field evidence for transfer of plastic debris along a terrestrial food chain. *Scientific Reports*, *7*(1). doi:10.1038/s41598-017-14588-2
5. English, M. D., Robertson, G. J., Avery-Gomm, S., Pirie-Hay, D., Roul, S., Ryan, P. C., … Mallory, M. L. (2015). Plastic and metal ingestion in three species of coastal waterfowl wintering in Atlantic Canada. *Marine Pollution Bulletin*, *98*(1–2), 349–353. doi:10.1016/j.marpolbul.2015.05.063
6. Bahrani, F., Mohammadi, A., Dobaradaran, S., De-la-Torre, G. E., Arfaeinia, H., Ramavandi, B., … Tekle-Röttering, A. (2024). Occurrence of microplastics in edible tissues of livestock (cow and sheep). *Environmental Science and Pollution Research International*, *31*(14), 22145–22157. doi:10.1007/s11356-024-32424-9
7. Susanti, R., Yuniastuti, A., & Fibriana, F. (2021). The evidence of microplastic contamination in central Javanese local ducks from intensive animal husbandry. *Water, Air, and Soil Pollution*, *232*(5). doi:10.1007/s11270-021-05142-y
8. Leslie DI van der VDLM van MMJM van VQRGDHA. Environment & Health Plastic Particles in Livestock Feed , Milk , Meat and Blood. 2022;(April)

**Plant-based food**

1. Aydın, R. B., Yozukmaz, A., Şener, İ., Temiz, F., & Giannetto, D. (2023). Occurrence of microplastics in most consumed fruits and vegetables from Turkey and public risk assessment for consumers. *Life (Basel, Switzerland)*, *13*(8). doi:10.3390/life13081686
2. Canha, N., Jafarova, M., Grifoni, L., Gamelas, C. A., Alves, L. C., Almeida, S. M., & Loppi, S. (2023). Microplastic contamination of lettuces grown in urban vegetable gardens in Lisbon (Portugal). *Scientific Reports*, *13*(1), 14278. doi:10.1038/s41598-023-40840-z
3. Battaglini, E., Miralles, P., Lotti, N., Soccio, M., Fiorini, M., & Coscollà, C. (2024). Analysis of microplastics in commercial vegetable edible oils from Italy and Spain. *Food Chemistry*, *443*(138567), 138567. doi:10.1016/j.foodchem.2024.138567
4. Milne, M. H., De Frond, H., Rochman, C. M., Mallos, N. J., Leonard, G. H., & Baechler, B. R. (2023). Exposure of U.S. adults to microplastics from commonly-consumed proteins. *Environmental Pollution (Barking, Essex: 1987)*, 123233. doi:10.1016/j.envpol.2023.123233
5. Walkinshaw, C., Tolhurst, T. J., Lindeque, P. K., Thompson, R., & Cole, M. (2022). Detection and characterisation of microplastics and microfibres in fishmeal and soybean meal. *Marine Pollution Bulletin*, *185*(Pt A), 114189. doi:10.1016/j.marpolbul.2022.114189

**Human samples**

1. Horvatits, T., Tamminga, M., Liu, B., Sebode, M., Carambia, A., Fischer, L., … Fischer, E. K. (2022). Microplastics detected in cirrhotic liver tissue. *EBioMedicine*, *82*(104147), 104147. doi:10.1016/j.ebiom.2022.104147
2. Schwabl, P., Köppel, S., Königshofer, P., Bucsics, T., Trauner, M., Reiberger, T., & Liebmann, B. (2019). Detection of various microplastics in human stool. *Annals of Internal Medicine*, *171*(7), 453–457. doi:10.7326/m19-0618

3. Wu, D., Feng, Y., Wang, R., Jiang, J., Guan, Q., Yang, X., … Luo, Y. (2023). Pigment microparticles and microplastics found in human thrombi based on Raman spectral evidence. *Journal of Advanced Research*, *49*, 141–150. doi:10.1016/j.jare.2022.09.004
4. Yan, Z., Liu, Y., Zhang, T., Zhang, F., Ren, H., & Zhang, Y. (2022). Analysis of microplastics in human feces reveals a correlation between fecal microplastics and inflammatory bowel disease status. *Environmental Science & Technology*, *56*(1), 414–421. doi:10.1021/acs.est.1c03924
5. Li, Zhiming, Wang, J., Gao, X., Du, J., Sui, H., Wu, J., … Huang, Z. (2023). Investigation of microplastics (≥10 μm) in meconium by Fourier transform infrared microspectroscopy. *Toxics*, *11*(4), 310. doi:10.3390/toxics11040310
6. Ibrahim, Y. S., Tuan Anuar, S., Azmi, A. A., Wan Mohd Khalik, W. M. A., Lehata, S., Hamzah, S. R., … Lee, Y. Y. (2021). Detection of microplastics in human colectomy specimens. *JGH Open : An Open Access Journal of Gastroenterology and Hepatology*, *5*(1), 116–121. doi:10.1002/jgh3.12457
7. Rotchell, J. M., Jenner, L. C., Chapman, E., Bennett, R. T., Bolanle, I. O., Loubani, M., … Palmer, T. M. (2023). Detection of microplastics in human saphenous vein tissue using μFTIR: A pilot study. *PloS One*, *18*(2), e0280594. doi:10.1371/journal.pone.0280594
8. Halfar, J., Čabanová, K., Vávra, K., Delongová, P., Motyka, O., Špaček, R., … Heviánková, S. (2023). Microplastics and additives in patients with preterm birth: The first evidence of their presence in both human amniotic fluid and placenta. *Chemosphere*, *343*(140301), 140301. doi:10.1016/j.chemosphere.2023.140301
9. Guan, Q., Jiang, J., Huang, Y., Wang, Q., Liu, Z., Ma, X., … Xia, Y. (2023). The landscape of micron-scale particles including microplastics in human enclosed body fluids. *Journal of Hazardous Materials*, *442*(130138), 130138. doi:10.1016/j.jhazmat.2022.130138
10. Yang, Y., Xie, E., Du, Z., Peng, Z., Han, Z., Li, L., … Yang, X. (2023). Detection of various microplastics in patients undergoing cardiac surgery. *Environmental Science & Technology*, *57*(30), 10911–10918. doi:10.1021/acs.est.2c07179
11. Ragusa, A., Notarstefano, V., Svelato, A., Belloni, A., Gioacchini, G., Blondeel, C., … Giorgini, E. (2022). Raman Microspectroscopy detection and characterisation of microplastics in human breastmilk. *Polymers*, *14*(13), 2700. doi:10.3390/polym14132700
12. Zhu, L., Kang, Y., Ma, M., Wu, Z., Zhang, L., Hu, R., … An, L. (2024). Tissue accumulation of microplastics and potential health risks in human. *The Science of the Total Environment*, *915*(170004), 170004. doi:10.1016/j.scitotenv.2024.170004
13. Ho, Y.-W., Lim, J. Y., Yeoh, Y. K., Chiou, J.-C., Zhu, Y., Lai, K. P., … Fang, J. K.-H. (2022). Preliminary findings of the high quantity of microplastics in Faeces of Hong Kong residents. *Toxics*, *10*(8), 414. doi:10.3390/toxics10080414
14. Liu, Sheng, Wang, C., Yang, Y., Du, Z., Li, L., Zhang, M., … Zhang, M. (2024). Microplastics in three types of human arteries detected by pyrolysis-gas chromatography/mass spectrometry (Py-GC/MS). *Journal of Hazardous Materials*, *469*(133855), 133855. doi:10.1016/j.jhazmat.2024.133855
15. Massardo, S., Verzola, D., Alberti, S., Caboni, C., Santostefano, M., Eugenio Verrina, E., … Artini, C. (2024). MicroRaman spectroscopy detects the presence of microplastics in human urine and kidney tissue. *Environment International*, *184*(108444), 108444. doi:10.1016/j.envint.2024.108444
16. Montano, L., Giorgini, E., Notarstefano, V., Notari, T., Ricciardi, M., Piscopo, M., & Motta, O. (2023). Raman Microspectroscopy evidence of microplastics in human semen. *The Science of the Total Environment*, *901*(165922), 165922. doi:10.1016/j.scitotenv.2023.165922
17. Liu, Shaojie, Liu, X., Guo, J., Yang, R., Wang, H., Sun, Y., … Dong, R. (2022). The association between microplastics and Microbiota in placentas and meconium: The first evidence in humans. *Environmental Science & Technology*. doi:10.1021/acs.est.2c04706
18. Weingrill, R. B., Lee, M.-J., Benny, P., Riel, J., Saiki, K., Garcia, J., … Urschitz, J. (2023). Temporal trends in microplastic accumulation in placentas from pregnancies in Hawaiʻi. *Environment International*, *180*(108220), 108220. doi:10.1016/j.envint.2023.108220
19. Zhang, N., Li, Y. B., He, H. R., Zhang, J. F., & Ma, G. S. (2021). You are what you eat: Microplastics in the feces of young men living in Beijing. *The Science of the Total Environment*, *767*(144345), 144345. doi:10.1016/j.scitotenv.2020.144345
20. Leslie, H. A., van Velzen, M. J. M., Brandsma, S. H., Vethaak, A. D., Garcia-Vallejo, J. J., & Lamoree, M. H. (2022). Discovery and quantification of plastic particle pollution in human blood. *Environment International*, *163*(107199), 107199. doi:10.1016/j.envint.2022.107199
21. Rotchell, J. M., Austin, C., Chapman, E., Atherall, C. A., Liddle, C. R., Dunstan, T. S., … Guinn, B.-A. (2024). Microplastics in human urine: Characterisation using μFTIR and sampling challenges using healthy donors and endometriosis participants. *Ecotoxicology and Environmental Safety*, *274*(116208), 116208. doi:10.1016/j.ecoenv.2024.116208

22. Li, Zhuo, Zheng, Y., Maimaiti, Z., Fu, J., Yang, F., Li, Z.-Y., … Xu, C. (2024). Identification and analysis of microplastics in human lower limb joints. *Journal of Hazardous Materials*, *461*(132640), 132640. doi:10.1016/j.jhazmat.2023.132640
23. Cetin, M., Demirkaya Miloglu, F., Kilic Baygutalp, N., Ceylan, O., Yildirim, S., Eser, G., & Gul, H. İ. (2023). Higher number of microplastics in tumoral colon tissues from patients with colorectal adenocarcinoma. *Environmental Chemistry Letters*, *21*(2), 639–646. doi:10.1007/s10311-022-01560-4
24. Xue, J., Xu, Z., Hu, X., Lu, Y., Zhao, Y., & Zhang, H. (2024). Microplastics in maternal amniotic fluid and their associations with gestational age. *The Science of the Total Environment*, *920*(171044), 171044. doi:10.1016/j.scitotenv.2024.171044
25. Zhong, Y., Yang, Y., Zhang, L., Ma, D., Wen, K., Cai, J., … Huang, Z. (2024). Revealing new insights: Two-center evidence of microplastics in human vitreous humor and their implications for ocular health. *The Science of the Total Environment*, *921*(171109), 171109. doi:10.1016/j.scitotenv.2024.171109
26. Zhao, Q., Zhu, L., Weng, J., Jin, Z., Cao, Y., Jiang, H., & Zhang, Z. (2023). Detection and characterization of microplastics in the human testis and semen. *The Science of the Total Environment*, *877*(162713), 162713. doi:10.1016/j.scitotenv.2023.162713
